# Copper mobilisation from Cu sulphide minerals by methanobactin: Effect of pH, oxygen and natural organic matter

**DOI:** 10.1111/gbi.12505

**Published:** 2022-06-18

**Authors:** Danielle D. Rushworth, Iso Christl, Naresh Kumar, Kevin Hoffmann, Ruben Kretzschmar, Moritz F. Lehmann, Walter D. C. Schenkeveld, Stephan M. Kraemer

**Affiliations:** ^1^ Centre for Microbiology and Environmental Systems Science University of Vienna Vienna Austria; ^2^ Soil Chemistry Institute of Biogeochemistry and Pollutant Dynamics, ETH Zurich Switzerland; ^3^ Soil Chemistry and Chemical Soil Quality, Environmental Sciences Wageningen University Wageningen The Netherlands; ^4^ Department of Environmental Geosciences University of Basel Basel Switzerland

**Keywords:** aerobic methane oxidation, chalkophore, Cu sulphide dissolution, ligand‐promoted dissolution, methanobactin, microbial Cu acquisition

## Abstract

Aerobic methane oxidation (MOx) depends critically on the availability of copper (Cu) as a crucial component of the metal centre of particulate methane monooxygenase, one of the main enzymes involved in MOx. Some methanotrophs have developed Cu acquisition strategies, in which they exude Cu‐binding ligands termed chalkophores under conditions of low Cu availability. A well‐characterised chalkophore is methanobactin (mb), exuded by the microaerophilic methanotroph *Methylosinus trichosporium* OB3b. Aerobic methanotrophs generally reside close to environmental oxic–anoxic interfaces, where the formation of Cu sulphide phases can aggravate the limitation of bioavailable Cu due to their low solubility. The reactivity of chalkophores towards such Cu sulphide mineral phases has not yet been investigated. In this study, a combination of dissolution experiments and equilibrium modelling was used to examine the dissolution and solubility of bulk and nanoparticulate Cu sulphide minerals in the presence of mb as influenced by pH, oxygen and natural organic matter. In general, we show that mb is effective at increasing the dissolved Cu concentrations in the presence of a variety of Cu sulphide phases that may potentially limit Cu bioavailability. More Cu was mobilised per mole of mb from Cu sulphide nanoparticles compared with well‐crystalline bulk covellite (CuS). In general, the efficacy of mb at mobilising Cu from Cu sulphides is pH‐dependent. At lower pH, e.g. pH 5, mb was ineffective at solubilizing Cu. The presence of mb increased dissolved Cu concentrations between pH 7 and 8.5, where the solubility of all Cu sulphides is generally low, both in the presence and absence of oxygen. These results suggest that chalkophore‐promoted Cu mobilisation from sulphide phases is an effective extracellular mechanism for increasing dissolved Cu concentrations at oxic–anoxic interfaces, particularly in the neutral to slightly alkaline pH range. This suggests that aerobic methanotrophs may be able to fulfil their Cu requirements via the exudation of mb in natural environments where the bioavailability of Cu is constrained by very stable Cu sulphide phases.

## 1 INTRODUCTION

Methane (CH_4_) emissions from terrestrial and marine environments contribute significantly to global warming, owing to its greater global warming potential (~25 times) than carbon dioxide (CO_2_) (Hartmann et al., 2013). After wetlands (70% of total natural emissions), marine and terrestrial environments contribute the most (>50% of the rest) to natural CH_4_ emissions (Kirschke et al., 2013). Methane oxidation by methane‐oxidising bacteria (MOB) or archaea (MOA) called methanotrophs decreases CH_4_ fluxes to the atmosphere (i.e. the biological methane filter). The aerobic oxidation of CH_4_ by bacteria (MOx) is catalysed by one of the two methane monooxygenase (MMO) enzymes; the copper (Cu)‐bearing particulate MMO (pMMO) or the iron (Fe)‐bearing soluble MMO (sMMO) (Hakemian et al., 2008; Hanson & Hanson, 1996). Although most MOB express pMMO, some can switch to expressing sMMO under Cu‐limiting conditions (Hakemian et al., 2008). In general, pMMO is more common than sMMO in natural environments and possesses a higher affinity for CH_4_ (Leak & Dalton, 1986; Murrell et al., 2000). Therefore, the efficiency of MOx relies directly on the availability of Cu in ambient waters and the capacity of methanotrophs to acquire Cu. In natural aquatic systems, however, the bioavailability of Cu can be very limited. For example, in freshwater systems, total Cu concentrations are found to be as low as 10^−9^ M, and free Cu^2+^ concentrations, which are considered a measure for readily bioavailable Cu, as low as 10^−16^ M have been observed (Xue & Sigg, 1993).

Because of their requirement for both CH_4_ and oxygen (O_2_), MOB generally reside near oxic–anoxic interfaces (Blees, Niemann, Wenk, Zopfi, Schubert, Kirf, et al., 2014b; Guggenheim et al., 2019; Hanson & Hanson, 1996; Rudd et al., 1976), often in close proximity to sulphidic waters (Lehmann, Bernasconi, Barbieri, et al., 2004; Lehmann, Bernasconi, McKenzie, et al., 2004). Dissolved sulphide can constrain free Cu^2+^ concentrations by forming thermodynamically stable Cu sulphide complexes and mineral phases, like covellite (Cu[I]S), which possess very low solubility products (Luther & Rickard, 2005; Luther & Tsamakis, 1989; Rickard & Luther, 2006; Shea & Helz, 1989). Dissolved Cu can also be incorporated into pyrite (FeS_2_), a mineral prevalent under sulphidic conditions, to form the Cu‐Fe sulphide mineral chalcopyrite (CuFeS_2_) (Cowper & Rickard, 1989). Once formed, Cu sulphides can traverse across oxic‐anoxic interfaces and are often observed in oxic waters (Kuwabara & Luther, 1993; Luther & Tsamakis, 1989; Rozan et al., 1999; Rozan et al., 2000) where they can persist for weeks, or even months (Sukola et al., 2005), despite their propensity towards oxidative dissolution. Slow oxidation kinetics of Cu sulphides (Millero et al., 1987) can further be inhibited at the low dissolved O_2_ concentrations observed in the vicinity of oxic‐anoxic interfaces. In eutrophic lakes, for example, O_2_ concentrations often decrease with depth in the water column from atmospheric‐equilibrium concentrations (>250 μM) in surface waters to sub‐micromolar concentrations within, and below, redox‐transition zones (Blees, Niemann, Wenk, Zopfi, Schubert, Kirf, et al., 2014b; Guggenheim et al., 2019). Despite these potentially limiting conditions, there is evidence of MOx occurring via pMMO in lakes where free Cu^2+^ concentrations are limited (Guggenheim et al., 2019).

It has been reported that some MOB cope with Cu limitation by exuding high‐affinity Cu‐binding organic ligands known as chalkophores (DiSpirito et al., 1998; Fitch et al., 1993; Kim et al., 2004), similar to the siderophores exuded by some plants and microorganisms for Fe acquisition (Kraemer, 2011; Kraemer et al., 2015). One such well‐characterised chalkophore is methanobactin (mb) exuded by the methanotroph *Methlyosinus trichosporium* OB3b (*M. trichosporium* OB3b) (Kim et al., 2005). From a biogeochemical perspective, mb facilitates Cu acquisition by increasing the dissolved Cu concentration through the formation of soluble Cu‐mb chelate complexes. While mb can also potentially complex other trace metals, e.g. Fe, Ni, Zn, Au, Ag, Pb, Mn, Cd and Co (Choi, Do, et al., 2006; McCabe et al., 2017), its high affinity for Cu enables it to effectively compete with other ligands in the environment (i.e. those comprised in the natural organic matter [NOM] pool) for complexing Cu (Pesch et al., 2013). The transfer of Cu between NOM constituents and mb occurs through fast ligand exchange reactions (Pesch et al., 2013). The equilibrium speciation of Cu‐mb complexes depends on the Cu to mb ratio and the pH; mb can form both 1:1 and 2:1 ligand to metal complexes, and depending on the pH, complexes can become protonated (Choi, Zea, et al., 2006; El Ghazouani et al., 2011; Pesch et al., 2012).

Several studies have reported on the physiological response of mb‐exuding MOB in the presence of Cu minerals, ranging from changes in enzyme expression (Fru et al., 2011; Knapp et al., 2007; Kulczycki et al., 2007) to changes in MOx rates (Kulczycki et al., 2007). It was previously shown that mb can increase Cu mobilisation from Cu‐doped borosilicate glass (Kulczycki et al., 2007). These studies have highlighted that Cu geochemistry influences methanotrophic activity. The reactivity of mb towards Cu‐bearing sulphide minerals that are likely to constrain Cu bioavailability in habitats where MOB reside remains to be elucidated.

The main goal of this study was to investigate Cu mobilisation from Cu sulphide phases by mb. Local environmental conditions, including the presence of NOM constituents, can affect various properties of sulphides in the environment, including particle size, particle aggregation state and thermodynamic stability (Deonarine & Hsu‐Kim, 2009; Hoffmann et al., 2020; Tiller & O'Melia, 1993). Therefore, we carried out batch dissolution experiments using both nanoparticulate Cu sulphides, including nanoparticles (NPs) synthesised in the presence or absence of fulvic acids, and well‐crystalline Cu sulphides, including synthetic covellite (CuS) and a natural chalcopyrite (CuFeS_2_) sample. Furthermore, to explore the geochemical constraints on Cu mobilisation from sulphides, we investigated the effect of both pH and O_2_ on Cu sulphide dissolution. A particular focus was Cu mobilisation in the neutral to slightly alkaline pH range, which is a characteristic of most natural freshwater and marine systems (Posacka et al., 2019) where bacterial copper limitation may occur. Considering the increasing stability of Cu‐mb complexes with increasing pH (Pesch et al., 2012), we hypothesised that mb‐promoted dissolution of Cu sulphides is effective in the neutral‐alkaline pH range and that this process accelerates Cu mobilisation even in the presence of O_2_, where sulphide oxidation provides an additional pathway of Cu mobilisation. Amorphous mineral phases are often characterised by lower thermodynamic stability and higher surface area compared with the corresponding more crystalline phases. Therefore, we hypothesised that the extent of Cu mobilisation by mb from Cu sulphides differs between sulphide phases, *specifically*, that more Cu is mobilised from poorly crystalline Cu_x_S NPs than from well‐crystalline CuS and CuFeS_2_. This work demonstrates that mb is part of an efficient Cu acquisition strategy employed by methanotrophs to overcome the geochemical constraints imposing Cu limitation.

## 2 MATERIALS AND METHODS

All solutions, unless otherwise specified, were prepared using ultra‐pure water (UPW) (18.2 MΩ cm, TOC < 2 ppb, Milli‐Q, Millipore). O_2_‐free UPW was prepared by boiling for ~1 h and then purging with N_2_ gas for 30 min, except for the preparation of Cu_x_S NPs where UPW was not boiled but purged with N_2_ for at least 2 h whilst stirring. All reagents used were analytical grade (Carl Roth, VWR, Sigma‐Aldrich) and glassware was presoaked in 1.4 M HNO_3_ (at least 24 h) and rinsed with UPW before use. Cellstar® centrifuge tubes were used as vessels for most experiments. Experiments were conducted in the dark to avoid photodegradation of the mb ligand and of NOM.

### 2.1 Methanobactin isolation

Cu‐free mb was isolated following previously established methods (Kim et al., 2005; Pesch et al., 2011). Briefly, *M. trichosporium* OB3b was incubated at 30°C in a 9 L fermenter containing a nitrate mineral salts (NMS) growth medium (Whittenbury et al., 1970) amended with 0.2 μM CuCl_2._ The fermenter was continuously purged with a mixture of air and CH_4_ that resulted in an O_2_:CH_4_ ratio of 4:1 (200 ml min^−1^). After filtration (0.22 μm cellulose filters, Millipore) and centrifugation, Cu‐free mb was isolated from the spent growth medium through a resin extraction (Diaion HP‐20, Supelco) using 60% methanol 40% UPW as a mobile phase. This was followed by a second purification step using reversed‐phase high‐pressure liquid chromatography (HPLC; Agilent 1260 Infinity bio‐inert system) whereby ~1.3 ml of crude material was injected into a Polaris 5 μm C18‐A 250 × 10.00 mm column and preguard using a mixed mobile phase of methanol and 10 mM NaCl in UPW (gradient outlined in Pesch et al., 2011). The eluent was monitored with a detector measuring UV–vis absorbance at 254 and 390 nm. Purified fractions were collated, rotary‐evaporated to remove methanol and lyophilized at the end of each day. The lyophilized product was redissolved in a small amount of UPW, and the concentration and purity of the product (82%–95%) were determined via the measurement of the C concentration and C/N ratio, respectively (Shimadzu TOC ‐LCPH analyser). The purity of the product was accounted for by diluting the product to achieve final Cu‐free mb stock concentrations of 1–2 mM. Stocks were stored at −20°C to avoid degradation.

### 2.2 Cu sulphide minerals

Cu sulphide nanoparticles (Cu_x_S NPs) were synthesised in the presence and absence of Suwannee River fulvic acid standard II (SRFA; Cat. No. 2S101F; IHSS) (5 mg C L^−1^) according to a procedure published earlier (Hoffmann et al., 2020). Synthesis was carried out in an anaerobic chamber by adding 50 μM CuCl_2_ and 100 μM Na_2_S·9H_2_O to a solution containing 10 mM NaCl and 1 mM 3‐morpholinopropane‐1‐sulphonic acid (MOPS) pH‐buffer (pH 7.5). To minimise possible effects from particle aggregation on dissolution kinetics, NPs were synthesised freshly before the start of the experiments. This ensured that NPs remained <10 nm in diameter for the duration of the experiment, as evidenced by the extensive characterisation of the Cu_x_S NPs previously reported (Hoffmann et al., 2020). Chemical speciation calculations outlined in Hoffmann et al. (2020) predict that 100% of the added Cu precipitates with sulphide, even in the presence of SRFA, and covellite (CuS) was the dominant mineral phase (Hoffmann et al., 2020). Assuming a spherical shape and based on the mean Feret diameter of the NPs, a specific surface area (SSA) of 133 m^2^ g^−1^ was estimated for Cu_x_S NPs synthesised using this method.

The well‐crystalline CuS was synthesised as previously described (Tezuka et al., 2007) with minor modifications. Briefly, 0.01 mol of powdered elemental sulphur (S^0^) and 0.01 mol of powdered elemental Cu (Cu^0^) were ground inside a nitrogen‐filled anaerobic chamber (mBRAUN, unilab 7185) using an agate pestle and mortar. The ground reagents were suspended in 20 ml O_2_‐free UPW in 50 ml Teflon tubes, sealed in pressure vessels and heated at 180°C for 72 h. The precipitate was collected inside the anaerobic chamber and was washed several times with O_2_‐free UPW before drying under N_2_. A natural chalcopyrite (CuFeS_2_) sample was purchased from Alfa Aesar. The phase purities of CuS and CuFeS_2_ were analysed using powder X‐ray diffraction (XRD, PANalytical X‐ray diffractometer [X'pert pro]). Rietveld refinements showed that the natural chalcopyrite sample contained ~72% CuFeS_2_, ~14.5% pyrite (FeS_2_) and ~7% of the phyllosilicate mineral chamosite (Fe_2_Al_2_SiO_5_) (Figure S1). No Cu‐bearing phases other than CuFeS_2_ were identified. The SSAs of CuS and CuFeS_2_ (0.58 and 0.78 m^2^ g^−1^, respectively) were determined using a multipoint N_2_−BET adsorption method (Quantachrome Nava 2000). To prevent oxidation, all minerals were stored in a N_2_ atmosphere until use.

### 2.3 Dissolution experiments

**FIGURE 1:**
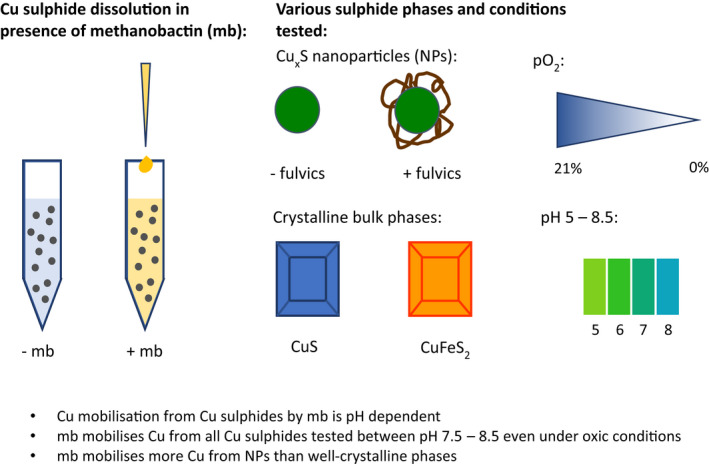
Schematic representation of the batch dissolution experiments used to investigate Cu mobilisation by mb from several Cu sulphide mineral phases and the conditions under which this was tested

In aquatic systems Cu sulphides are often found as nanoparticles (Hofacker et al., 2013; Weber, Voegelin, Kaegi, & Kretzschmar, 2009), which can interact with NOM, to which Cu can also be bound (Xue et al., 1996). Therefore, we investigated Cu mobilisation by mb from Cu_x_S NPs in the presence and absence of NOM. We also investigated Cu mobilisation by mb from crystalline CuS. To examine potential inhibitory effects caused by competitive complexation of Fe, Cu mobilisation from the mineral CuFeS_2_ was also investigated. Although concentrations of up to 50 μM mb have been observed in *M. trichosporium* OB3b laboratory cultures (Semrau et al., 2010), environmental concentrations are speculated to be as low as 0.5 μM (Pesch et al., 2013). To facilitate quantification of dissolved Cu concentrations approximately 20 μM mb was used in dissolution experiments. All the buffers used were selected for their very low affinity for Cu (Mash et al., 2003; Yu et al., 1997); therefore, they are not expected to affect Cu solution speciation in our experiments. All experiments were conducted at room temperature (20°C). Buffers were adjusted to the correct pH before the experiment using either HCl or NaOH. A schematic outline of the dissolution experiments is presented in Figure 1 along with the main findings from the study.

### 2.4 Cu_x_S nanoparticles dissolution

Aliquots of Cu_x_S NPs (pH 7.5) in UPW were amended with 19 μM mb in serum bottles under a N_2_ atmosphere (21 ml solution: 36 ml N_2_ headspace). Final total Cu, S and SRFA (where applicable) concentrations after amendment with mb were 47.5 μM, 95 μM and 4.75 mg C L^−1^, respectively. However, because CuS is the dominant phase formed, half of the sulphide is estimated to remain in solution or in the gas phase. We cannot exclude that traces of elemental sulphur or polysulphide formed. Yet, once formed they are quite stable and are unlikely to affect Cu mobilisation. Therefore, they were not considered in our experimental interpretations. With the given synthesis protocol, the expected NP suspension density was 4.5 mg L^−1^ (±5%) for all pH conditions. Control experiments with an equivalent background solution, but without mb amendment, were also included for comparison. Bottles were sealed with gas‐tight stoppers and were kept on a continuous lateral shaker (~200 rpm). Periodic sampling involved a coagulation step in which 0.5 ml of the Cu_x_S NP suspension (taken using a gas‐tight syringe) was added to a 4.5 ml CaCl_2_ solution (1 mM final concentration) and shaken/mixed for 2 min. The suspension was then filtered (0.22 μm Nylon filters) to separate coagulated NPs from solution, and an aliquot of the remaining filtrate was acidified (1% HNO_3_) and preserved (4°C) for analysis of the total dissolved Cu concentration. The coagulation and filtration method was verified in previous work as a suitable method for separating NPs from the filtrate (Hoffmann et al., 2020). To examine the effect of pH on Cu_x_S NPs dissolution by mb, sub‐sets from the Cu_x_S NP suspensions were adjusted using buffer solutions adjusted to pH 5 using N,N′‐diethylpiperazine (DEPP) buffer, pH 6 using 2‐(N‐morpholino)ethanesulphonic acid (MES) buffer and pH 8.5 using DEPP. The resulting concentrations of reactants and electrolytes slightly differ from the experiments at pH 7.5 and are outlined in Table S1. The speciation of sulphide is pH‐dependent, therefore, the species distribution of Cu‐free sulphide at equilibrium for all experimental pH conditions was quantified using Henry's law (Table S1). The effect of pO_2_ on Cu mobilisation by mb was examined by adjusting the O_2_ concentration in the headspace to 0.01 and 0.21 atm. It was previously demonstrated that Cu_x_S NPs are highly resistant to oxidative dissolution under oxic conditions (Hoffmann et al., 2020). However, NOM is ubiquitous in natural systems and it was shown by the same authors that oxidative dissolution in fact does occur in the presence of SRFA. It is already known that mb efficiently scavenges Cu from NOM complexes (Pesch et al., 2013), rendering it bioavailable for methanotrophs. Here, we investigated whether the exudation of mb could afford the additional benefit to further catalyse the dissolution process. Therefore, Cu mobilisation from Cu_x_S NPs by mb under oxic conditions was investigated for NPs synthesised with SRFA only and supported with equilibrium predictions to determine, whether any increase in dissolved Cu concentration is a result of Cu‐mb complex formation. From here on, Cu_x_S NP suspensions treated with 0.01 atm and 0.21 atm O_2_ will be referred to as ‘low O_2_’ and ‘high O_2_’ conditions, respectively. To adjust the O_2_ concentration, immediately after the Cu_x_S NPs were amended with mb, a specific volume of N_2_ gas in the headspace was replaced with pure O_2_ using a gas‐tight syringe and serum bottles treated as they were under anoxic conditions. The supplied amount of O_2_ in both the 0.01 and 0.21 atm O_2_ treatments exceeded the amount required to oxidise all the sulphide in the reactor (including solid + dissolved + gas phase sulphide) to sulphate, by a factor of 1.85 and 38.65, respectively. The pH was monitored and remained within ±0.15 units from the set pH value throughout all the experiments.

### 2.5 CuS and CuFeS_2_ dissolution

Cu mobilisation from well‐crystalline CuS and CuFeS_2_ by mb was examined in batch experiments at pH 6 to 8.5 (in 0.5 pH unit increments) under anoxic conditions in an anaerobic chamber. The pH was maintained using the pH buffers MES (pH 6 and 6.5), MOPS (pH 7 and 7.5) and piperazine‐N,N′‐*bis*(3‐propane sulphonic acid) (PIPPS) (pH 8 and 8.5). Duplicate mineral suspensions (2 g L^−1^ powdered mineral) were prepared in a 10 mM NaCl and 10 mM buffer solution using deoxygenated UPW. An mb ligand dose (20 μM) was added to the mineral suspensions, and control suspensions without the mb addition were also prepared. Suspensions were shaken continuously on an end‐over‐end shaker (16 rpm). The effect of O_2_ on Cu mobilisation from well‐crystalline CuS and CuFeS_2_ by mb was examined by exposing mineral suspensions to atmospheric pO_2_ (0.21 atm) immediately after the addition of mb (*t* = 0). The reactors (non‐gas‐tight centrifuge tubes) were then capped and periodically exposed to the atmosphere during sampling. The pH was monitored throughout the experiments and remained within ±0.1 pH units from the set pH values. Samples were taken at predetermined times, filtered through 0.22 μm PVDF membranes, acidified (1% HNO_3_) and stored (4°C) until total dissolved metal concentration analysis.

### 2.6 Analytics

Total dissolved metal concentrations were measured using either inductively coupled plasma optical emission spectrometry (ICP‐OES) (Perkin Elmer Optima 5300‐DV) or inductively coupled plasma mass spectrometry (ICP‐MS) (Agilent‐7700). Solution pH was monitored using a pH metre (Orion 3 star, Thermo).

### 2.7 Thermodynamic modelling

Visual MINTEQ Ver. 3.1 was used to predict Cu speciation in the oxic experimental Cu_x_S NP suspensions. The ‘non‐ideal consistent competitive adsorption' (NICA)‐Donnan model (Kinniburgh et al., 1996), using the generic parameters for Cu and proton binding to fulvic acids (Table S2) (Milne et al., 2003), was included for Cu_x_S NP speciation calculations to account for Cu binding to SRFA. Databases comp_2008.vdb; thermo.vdb and type6.vdb gaussian.vdb were used for calculations made in Visual MINTEQ. Due to its presence in the natural mineral CuFeS_2_, the solubility of Fe(II)‐bearing chamosite was investigated using the modelling programme PHREEQC. Fe(II) is the only constituent present in chamosite that may interact with mb, therefore, it was only necessary to assess the solubility of Fe. The MINTEQ v4 database (Parkhurst & Appelo, 2013) supplemented with the solubility constant for chamosite from the LLNL (Lawrence Livermore National Laboratory) database was used. Possible secondary mineral phases following the well‐crystalline sulphide phase oxidation were determined by allowing solutions to equilibrate with atmospheric pO_2_ and pCO_2_ (415 ppm). The influence of pH on the solubility of secondary phases was also determined and compared with data from oxic dissolution experiments. The effect of mb on total dissolved Cu concentrations in equilibrium with secondary phases was also predicted. Input parameters for Visual MINTEQ and PHREEQC are presented in Tables S3 and S4, respectively. Databases were complemented with protonation and Cu complexation constants for mb (Pesch et al., 2012).

## 3 RESULTS

### 3.1 Cu mobilisation from Cu_x_S nanoparticles

**FIGURE 2:**
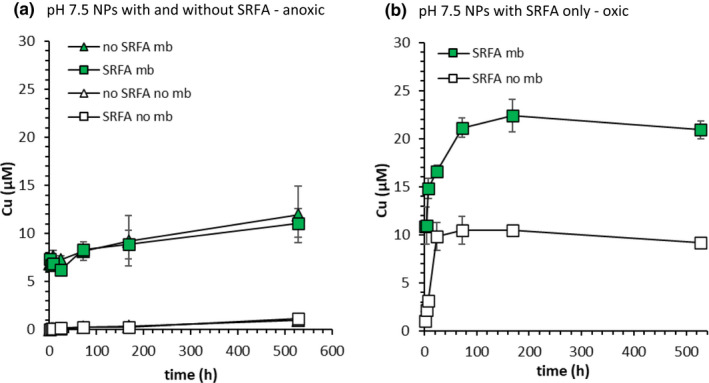
Cu mobilisation from Cu_x_S NPs (10 mM NaCl) in the presence of Suwannee River fulvic acids (SRFA) (squares; 5 mg C L^−1^) and absence of SRFA (triangles) at pH 7.5 under (a) anoxic conditions and (b) under oxic conditions (pO_2_ = 0.21 atm). Filled symbols show mb treatments (19 μM mb), and open symbols show controls (no mb). Error bars represent the range of duplicate measurements

Under anoxic conditions at pH 7.5, total dissolved Cu concentrations in the presence of Cu_x_S NPs reached a maximum of only 1.1 μM (528 h; 22 d) in absence of mb (Figure 2a). Upon addition of 19 μM mb dissolved Cu concentrations increased to 7.0 μM after only 1 h, and to a maximum of 12.0 μM after 528 h. While SRFA alone did not increase dissolved Cu concentrations under anoxic conditions, Cu mobilisation in the presence of mb was fast (Figure 2a).

**FIGURE 3:**
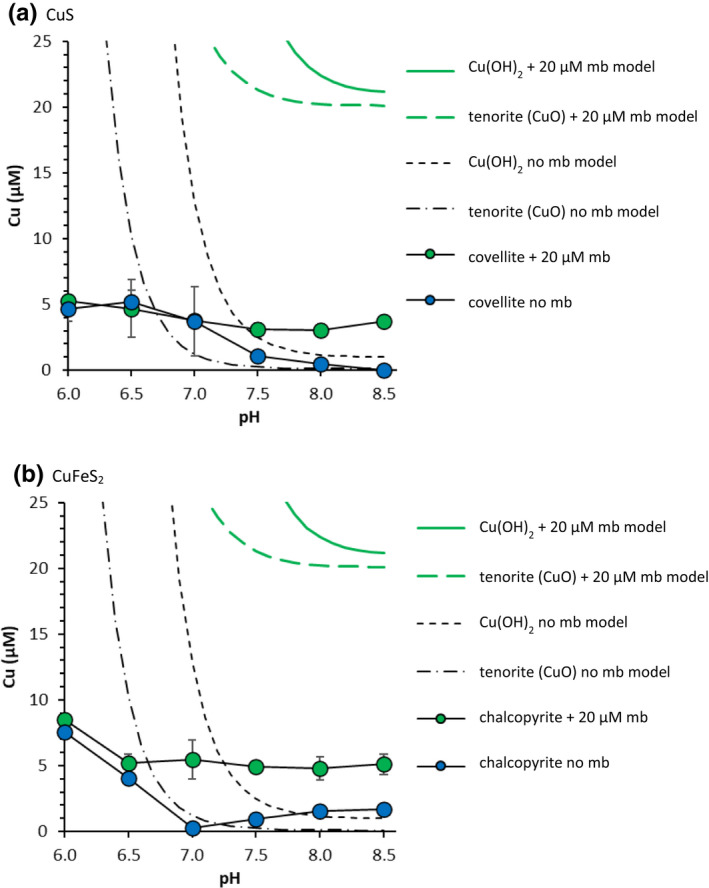
Cu concentrations mobilised from well‐crystalline (a) covellite and (b) chalcopyrite after 8 hours under oxic conditions in the presence and absence of 20 μM mb as a function of pH (2 g L^−1^ solids, 10 mM NaCl). The dashed black lines show the Cu concentration in equilibrium with Cu(OH)_2_ and tenorite (CuO) without mb, and the solid and dashed green lines show the Cu concentration in equilibrium with Cu(OH)_2_ and tenorite, respectively, plus 20 μM mb as a function of pH, calculated using PHREEQC (10 mM NaCl)

Under high O_2_ conditions at pH 7.5, even in the absence of mb, dissolved Cu concentrations increased rapidly to 9 μM (Figure 2b). Interestingly, the presence of mb and O_2_ had an additive effect on dissolved Cu concentrations at pH 7.5. The final dissolved Cu concentration for Cu_x_S NPs with SRFA and mb (20.9 μM; Figure 2b) was approximately equal to the combined final concentration for Cu_x_S NPs with mb but without O_2_ (11.1 μM; Figure 2a), and Cu_x_S NPs with O_2_ but without mb (9.2 μM; Figure 2b). As shown by the equilibrium calculations (Table S3), dissolved Cu concentrations are limited in the presence of O_2_ by the formation of secondary Cu (hydr)oxide mineral phases like Cu(OH)_2_ or CuO (tenorite). Although Cu(OH)_2_ is thermodynamically less stable than CuO and, therefore, expected to precipitate first, dissolved Cu concentrations in equilibrium with Cu(OH)_2_ (Figure 3) are, at pH 6 for example, up to 3 orders of magnitude higher than those observed experimentally below pH 7. Therefore, it is likely that dissolved Cu concentrations are constrained by a thermodynamically more stable phase, like CuO, or by very slow oxidation kinetics. In the presence of SRFA, predicted total Cu‐SRFA concentrations increase to micromolar concentrations in the presence of O_2_ (Table S3). Although Cu‐SRFA complexes form in the presence of O_2_, Cu‐mb complexes are still predicted to dominate the dissolved Cu speciation when mb is present. For example, based on predictions, 19.0 μM of the 26.0 μM dissolved Cu in equilibrium with crystalline CuO (pH 7.5) is attributed to Cu‐mb complexes (Table S3).

Cu mobilisation from Cu_x_S NPs was also examined under low O_2_ conditions (Figure S3). SRFA alone had a larger effect on Cu mobilisation at pH 7.5 under low O_2_ (0.01 atm) conditions than under high O_2_ conditions (Figure S2). Initial Cu mobilisation rates, i.e. Cu concentrations mobilised after 1 h, under low O_2_ conditions (6.2 μM Cu h^−1^) were more than 6 times those under high O_2_ conditions (1.0 μM Cu h^−1^) (Figure S2). Under low O_2_ conditions in Cu_x_S NP suspensions with SRFA, the final (528 h only) dissolved Cu concentrations were comparable in the presence and absence of mb. This result stands in contrast to the results observed under anoxic conditions and high O_2_ conditions (Figure 2), where the addition of mb increased dissolved Cu concentrations by 10.0 μM and 11.7 μM, respectively, for Cu_x_S NPs with SRFA.

Our results also suggest that the pH influenced the extent of Cu mobilisation from Cu_x_S NPs by mb, under both anoxic and low O_2_ conditions (Figure S3). Cu_x_S NP dissolution was particularly slow at pH 5 and 8.5 in the absence of mb under anoxic conditions, and total dissolved Cu concentrations reached only 1.6 and 0.9 μM at pH 5 and 8.5, respectively, after 168 h (Figure S3a). Under anoxic conditions dissolved Cu concentrations increased in presence of mb relative to the corresponding control at all examined pH except pH 5, where dissolved Cu concentrations remained below 1.7 μM (Figure S3a). Under low O_2_ conditions, Cu_x_S NPs dissolved at all examined pH values, but the addition of mb to Cu_x_S NP suspensions only enhanced Cu mobilisation at pH 6 and 8.5 (Figure S3b). At pH 5, 7.5 and 8.5 in absence of mb, dissolved Cu concentrations were higher under low O_2_ conditions than under anoxic conditions (Figure S3a,b).

### 3.2 Effect of pH on Cu (and Fe) mobilisation from CuS and CuFeS_2_ under anoxic conditions

**FIGURE 4:**
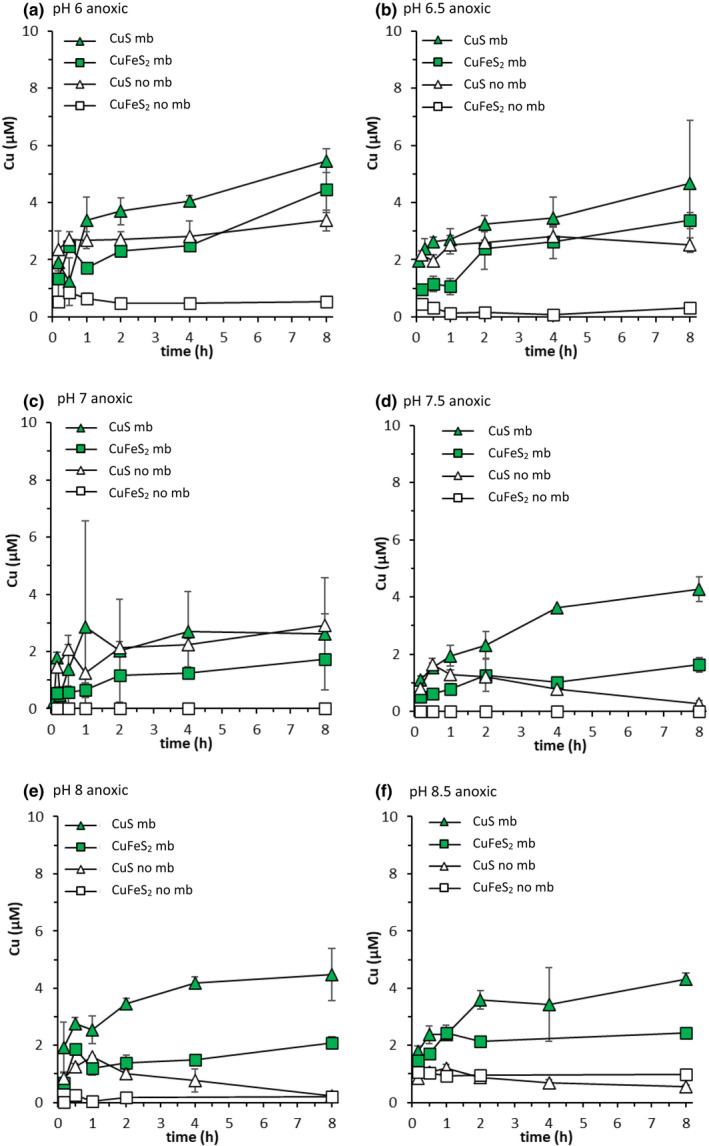
Cu mobilisation from covellite (triangles) and chalcopyrite (squares) (both 2 g L^−1^, 10 mM NaCl) under anoxic conditions at (a) pH 6 (10 mM MES buffer), (b) 6.5 (10 mM MES buffer), (c) pH 7 (10 mM MOPS buffer), (d) pH 7.5 (10 mM MOPS buffer), (e) pH 8 (10 mM PIPPS buffer) and (f) pH 8.5 (10 mM PIPPS buffer). Filled symbols show mb treatment (20 μM mb), and open symbols show controls (no mb). Error bars represent the range of duplicate measurements (except at pH 7 CuS mb; *n* = 4)

The effect of mb on Cu (and Fe) mobilisation from well‐crystalline CuS and CuFeS_2_ was investigated for the pH range 6–8.5. In the absence of mb, dissolved Cu concentrations were <1.1 μM in CuFeS_2_ suspensions at all pH values (Figure 4), but in CuS suspensions, they were larger at some pH values (pH 6–7; Figure 4), with a maximum concentration at pH 6 (3.4 μM; Figure 4a). Although dissolved Cu concentrations in CuS suspensions initially increased in absence of mb at higher pH values (Figure 4), they decreased to <0.6 μM after 8 h. Above pH 7, final dissolved Cu concentrations below 1.0 μM were observed for both minerals.

**FIGURE 5:**
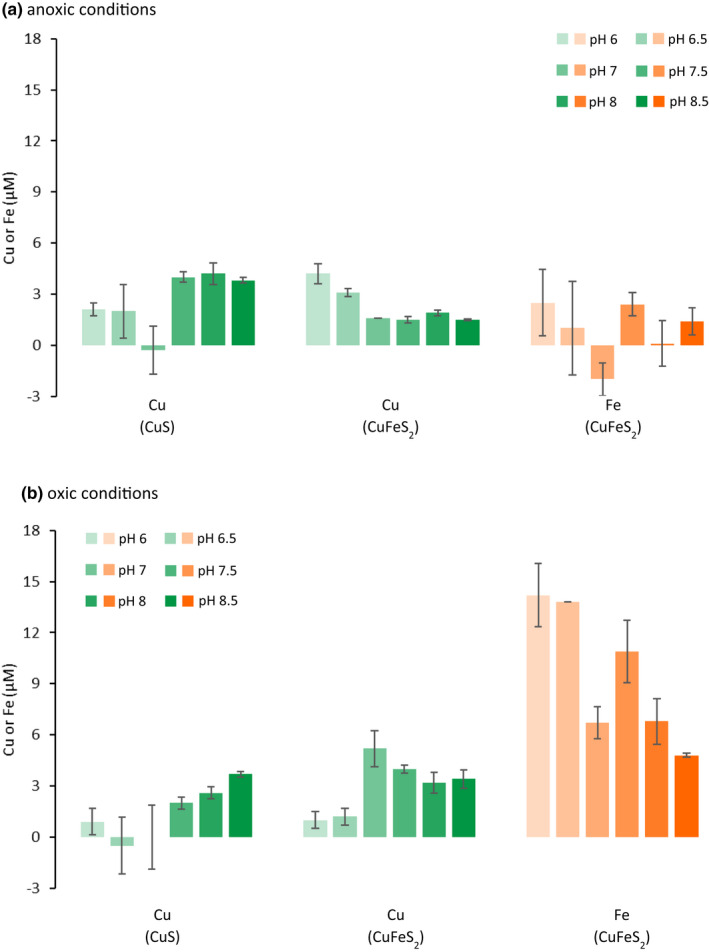
Final (8 h) Cu (green) and Fe (orange) concentrations (μM) in the presence of mb minus the control without mb in CuS and CuFeS_2_ dissolution experiments under (a) anoxic conditions and (b) oxic conditions from pH 6 to pH 8.5 (colour gradient increases with increasing pH). The data shown correspond to those presented in Figures 3, 6, S4 and S5 for Cu and Figures 5, 7 and S6 for Fe. Error bars represent the range of duplicate measurements

At most pH values, for both CuS and CuFeS_2_, the addition of mb increased Cu mobilisation relative to controls (Figures 4 and 5). The highest dissolved Cu concentrations were observed after 8 h in the presence of mb at pH 6 (5.5 and 4.5 μM for CuS and CuFeS_2_, respectively; Figure 4a). The lowest final dissolved Cu concentrations (8 h) in presence of mb were observed at pH 7 for CuS (2.6 μM) and at pH 7.5 for CuFeS_2_ (1.6 μM) (Figure 4c, d). The enhancing effect of mb on the Cu concentration, i.e. the difference in final dissolved Cu concentrations between corresponding mb and control treatments, was influenced not only by pH but also by the mineral type (Figure 5). For CuFeS_2_, mb had the greatest enhancing effect on Cu mobilisation at pH 6–7 whereas for CuS mb was most effective for Cu mobilisation at pH 7.5–8.5 (Figures 4a–c and 5). For CuFeS_2_ the effect was largest at pH 6 (4.2 μM) and for CuS at pH 8 (4.2 μM).

**FIGURE 6:**
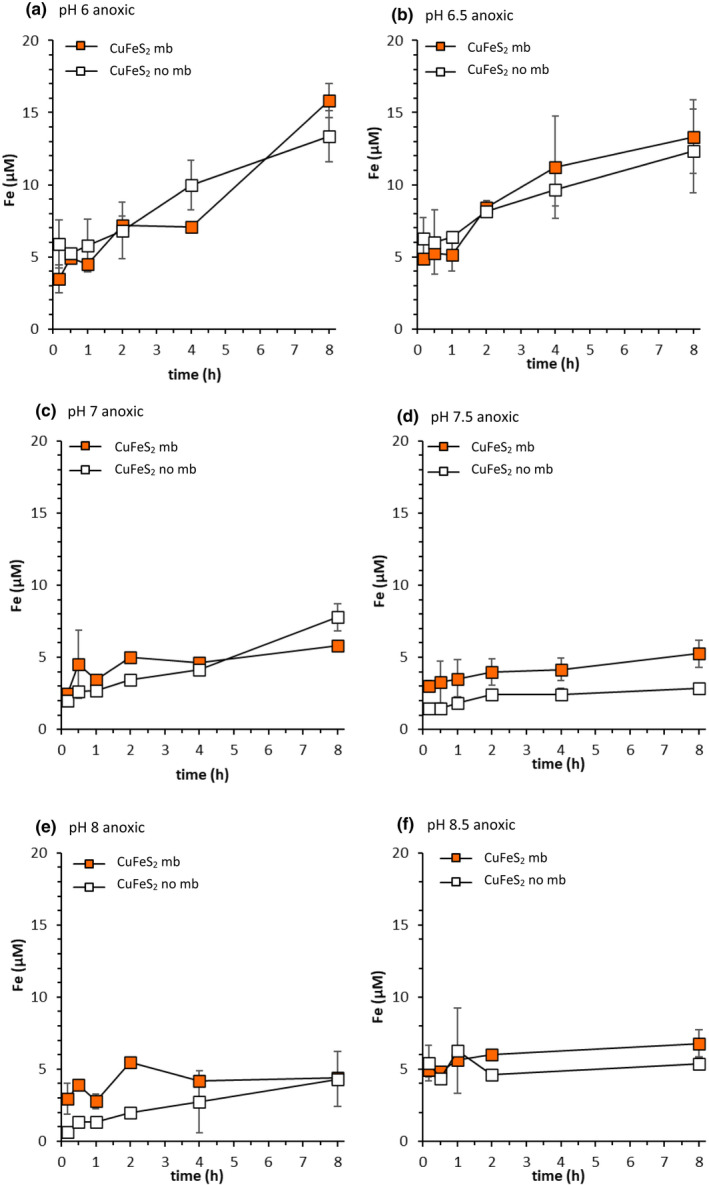
Fe mobilisation from chalcopyrite (2 g L^−1^, 10 mM NaCl) under anoxic conditions at (a) pH 6 (10 mM MES buffer), (b) 6.5 (10 mM MES buffer), (c) pH 7 (10 mM MOPS buffer), (d) pH 7.5 (10 mM MOPS buffer), (e) pH 8 (10 mM PIPPS buffer) and (f) pH 8.5 (10 mM PIPPS buffer). Filled symbols show mb treatments (20 μM mb), and open symbols show controls (no mb). Error bars represent the range of duplicate measurements

Dissolved Fe concentrations increased over time in CuFeS_2_ suspensions, except at pH 8.5 (Figure 6). Also, dissolved Fe concentrations decreased with increasing pH between pH 6–7.5 in the absence of mb (Figure 6a–d). The addition of 20 μM mb had no significant effect on dissolved Fe concentrations relative to controls (absence of mb), except at pH 7.5 and pH 8 (Figure 6 and Table S5). The increase in dissolved Fe concentration between the first time point (10 min) and the last (8 h) was smaller at higher pH values (i.e. pH 7.5–8.5). The highest dissolved Fe concentration (15.8 μM; at 8 h) was observed at pH 6 in the presence of mb; the lowest (2.8 μM; at 8 h) at pH 7.5 in absence of mb (Figure 6). The sum of Cu and Fe concentrations mobilised from CuFeS_2_ in presence of mb minus the sum of Cu and Fe concentrations in absence of mb (Figure 5) did not exceed the added mb concentration at any pH.

### 3.3 Effect of oxygen on Cu (and Fe) mobilisation from CuS and CuFeS_2_

**FIGURE 7:**
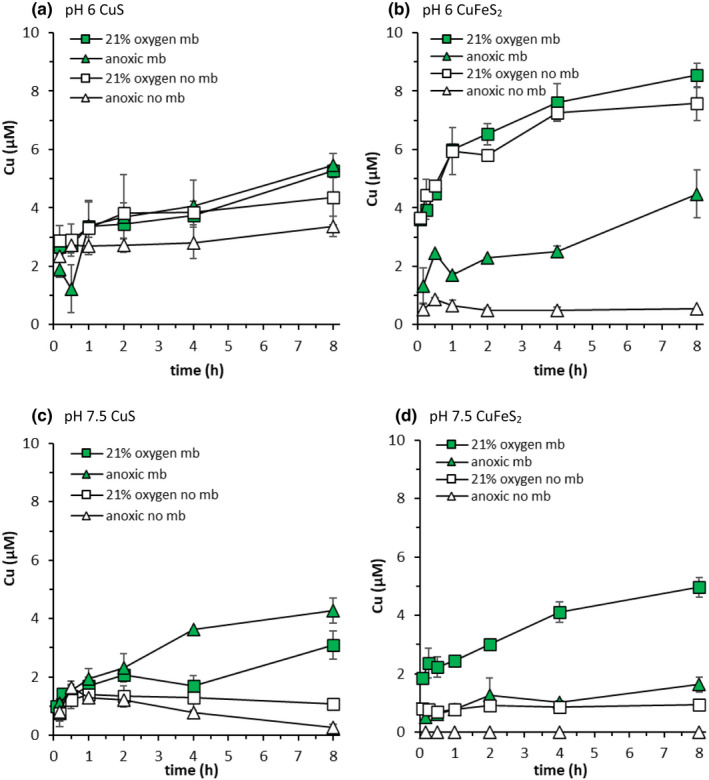
Cu mobilisation from covellite (a and c) and chalcopyrite (b and d) (both 2 g L^−1^, 10 mM NaCl) under oxic conditions (squares; pO_2_ = 0.21 atm) and under anoxic conditions (triangles) at pH 6 (left; 10 mM MES buffer) and pH 7.5 (right; 10 mM MOPS buffer). Filled symbols show mb treatments (20 μM mb), and open symbols show controls (no mb). Error bars represent the range of duplicate measurements. Anoxic data are the same as data shown in Figure 3

The effect of O_2_ on Cu mobilisation from CuS and CuFeS_2_ by mb was investigated for the pH range 6–8.5 (Figures 5b, 7, S4 and S5). At pH 6, the effect of mb on dissolved Cu concentrations relative to controls was much greater under anoxic conditions than under oxic conditions (Figures 5 and 7a, b). Although more Cu was mobilised from CuS than from CuFeS_2_ under anoxic conditions in absence of mb, the enhancing effect of mb on dissolved Cu concentrations was greater for CuFeS_2_. In CuS suspensions with added mb, final dissolved Cu concentrations were approximately the same under anoxic conditions (5.5 μM Cu) as they were in the presence of O_2_ (5.3 μM Cu). For CuFeS_2_, though, dissolved Cu concentrations reached 8.5 μM under oxic conditions in the presence of mb, as opposed to 4.5 μM under anoxic conditions (Figure 7b).

The addition of mb had a significantly greater effect on Cu mobilisation in the presence of O_2_ at pH 7.5 than at pH 6 (Figure 7c, d). Final dissolved Cu concentrations increased by 2 μM for CuS and 4 μM for CuFeS_2_ in presence of mb relative to controls at pH 7.5 (Figure 5). In similarity to pH 6, mb had a greater effect on final dissolved Cu concentrations, relative to the controls, under anoxic conditions (4.0 μM Cu) than under oxic conditions (2.0 μM) for CuS at pH 7.5 (Figure 5). For CuFeS_2_ at pH 7.5, however, mb had the greatest effect on dissolved Cu concentrations relative to controls under oxic conditions (4.0 μM), not under anoxic conditions (1.6 μM) (Figure 5). In the presence of O_2_, the enhancing effect of mb on mobilised Cu concentrations was smaller at the lower end of the examined pH range (pH 6) than at the higher end (pH 8.5) for both minerals (Figure 5).

**FIGURE 8:**
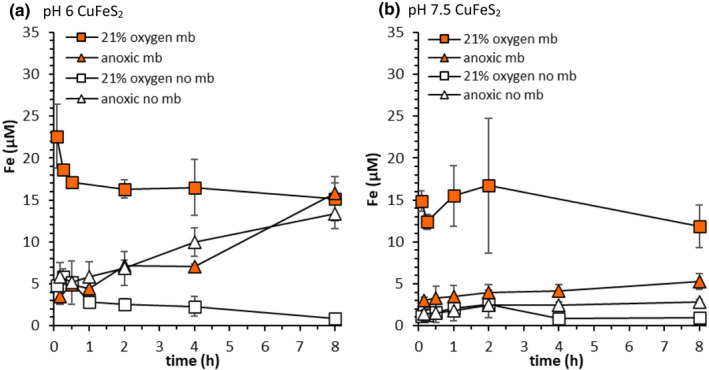
Fe mobilisation from CuFeS_2_ (2 g L^−1^, 10 mM NaCl) under oxic conditions (squares; pO_2_ = 0.21 atm) and under anoxic conditions (triangles) at pH 6 (left; 10 mM MES buffer) and pH 7.5 (right; 10 mM MOPS buffer). Filled symbols show mb treatments (20 μM mb), and open symbols show controls (no mb). Error bars represent the range of duplicate measurements. Anoxic data are the same as data shown in Figure 5

Fe mobilisation was also monitored during CuFeS_2_ dissolution under oxic conditions (Figures 8 and S6). While a contribution from nanoparticulate Fe(hydr)oxide minerals to dissolved Fe concentrations cannot be excluded, any contributions are expected to be minimal, and, dissolved Fe concentrations in absence of mb were much lower under oxic conditions than anoxic conditions. Methanobactin increased dissolved Fe concentrations significantly under oxic conditions at most pH values, whereas it had little effect under anoxic conditions (Figures 8 and S6). While the addition of mb did not increase dissolved Cu concentrations much relative to controls at pH 6 in the presence of O_2_, final dissolved Fe concentrations increased by 15 μM (Figure 5b). In the presence of mb under oxic conditions, the sum of the final dissolved Cu and Fe concentrations mobilised from CuFeS_2_ at pH 6 exceeded the added mb concentration. However, subtracting the Cu and Fe concentrations mobilised in the corresponding controls renders the sum of final dissolved Cu and Fe concentrations mobilised from CuFeS_2_ in presence of mb less than the added mb concentration (Figure 5b). At pH 7.5 under oxic conditions, the sum of the final dissolved Cu and Fe concentrations in presence of mb was approximately the same as the added mb concentration. Initial Fe mobilisation rates in the presence of mb were much larger under oxic conditions than anoxic conditions. Fe concentrations mobilised by mb were also influenced by pH. That is, in both the presence and absence of O_2_, Fe concentrations mobilised in presence of mb were highest under mildly acidic conditions (pH 6 and 6.5; Figures 5 and 8 and S6). For example, initial dissolved Fe concentrations in presence of mb were 22.6 μM at pH 6 and 14.9 μM at pH 7.5 under oxic conditions (Figure 8).

## 4 DISCUSSION

### 4.1 Cu_x_S nanoparticle solubility in the presence of mb

Cu_x_S NPs are found to persist under reducing conditions in both laboratory (Hoffmann et al., 2020; Kent & Vikesland, 2016) and field studies (Weber, Voegelin, Kaegi, & Kretzschmar, 2009; Weber, Voegelin, & Kretzschmar, 2009). Cu_x_S NPs, in absence of any mobilising/complexing agent, therefore, are likely to limit the bioavailability of Cu. Dissolved Cu concentrations under anoxic conditions also remained limited in Cu_x_S NP suspensions with SRFA (Figure 2a). Although SRFA can form complexes with Cu (Averett et al., 1994; Cabaniss & Shuman, 1988), the results presented here indicate that SRFA cannot substantially increase the dissolved Cu pool in the presence of sulphides. Based on this, we suggest that the majority of Cu is likely to remain in the solid phase in sulphidic environments independent of the availability of NOM. In microbial Cu acquisition strategies based on exudation of ligands like mb, the ligands must compete with sulphides for binding Cu in environments where sulphides influence Cu speciation. Enhanced Cu mobilisation from Cu_x_S NPs by mb at pH 6, 7.5 and 8.5 under anoxic conditions demonstrates that mb is an effective competing ligand for Cu that could help MOB to overcome Cu limitation at circumneutral pH in such environments (Figure 2a and S3a).

During complete oxidation of Cu sulphide, sulphide is oxidised to sulphate (${\mathrm{SO}}_{4}^{2-}$) and Cu^2+^ ions are released into the solution, as in the following equation for the oxidative dissolution of CuS:

(1) $$\mathrm{CuS}+2O_{2}\to {\mathrm{Cu}}^{2+}+{\mathrm{SO}}_{4}^{2-}$$

Dissolved Cu concentrations in Cu_x_S NP suspensions at pH 7.5 in absence of mb and SRFA were low in the presence of oxygen (Figure S2a). This is consistent with the observation of Cu_x_S NPs in oxic freshwater environments (Rozan et al., 1999; Rozan et al., 2000). One reason for their persistence in the presence of O_2_ could be passivation of the mineral surface caused by oxidation of surface sulphur (S) species or of excess soluble sulphide used in the preparations of elemental sulphur (S^0^) (Li et al., 2017). Another possible reason could be that Cu_x_S NP dissolution is simply kinetically inhibited. At any rate, aerobic methanotrophs reside at oxic‐anoxic interfaces where O_2_ concentrations are low, often orders of magnitude lower than our lowest examined pO_2_ (Blees, Niemann, Wenk, Zopfi, Schubert, Jenzer, et al., 2014a; Blees, Niemann, Wenk, Zopfi, Schubert, Kirf, et al., 2014b). The exudation of mb by methanotrophs may be particularly important at these interfaces where the oxidative dissolution of Cu_x_S NPs traversing from sulphidic zones is likely to be extremely slow.

It has been shown that Cu_x_S NPs are stable in absence of SRFA and under oxic conditions (Hoffmann et al., 2020). Increased dissolved Cu concentrations in Cu_x_S NP suspensions with SRFA, and without mb under oxic conditions, compared with anoxic conditions (Figure 2), suggests that oxidation of Cu_x_S NPs can lead to complexation of Cu by SRFA. Additionally, the formation of Cu‐SRFA complexes is implied because the dissolved Cu concentration exceeds the predicted dissolved Cu concentration in equilibrium with Cu(OH)_2_ (Figure 3). Even though SRFA increases dissolved Cu concentrations, its impact on the bioavailability of Cu may be different from that of mb. One study found that some ligands, e.g. EDTA, greatly limited cell‐associated Cu concentrations in *M. trichosporium* OB3b cultures (Morton et al., 2000). In another study, Cu accumulation in *Methylomicrobium albus* BG8 cells was correlated with the free Cu^2+^ concentration in the growth media, not with total Cu concentrations, which was dominated by Cu‐EDTA complexes (Berson & Lidstrom, 1996). Both studies suggest that by no means all dissolved Cu in organic complexes is necessarily bioavailable. However, mb is speculated to serve several roles in methanotrophic Cu homeostasis, e.g. as an intracellular chaperone that assists in utilising Cu bound to Cu storage proteins (DiSpirito et al., 2016), and is taken up by specific transporters (Gu et al., 2016), making it a key component for methanotrophic Cu acquisition. Previous work in this context has shown that mb can chelate Cu from NOM complexes (Pesch et al., 2013). Hence, Cu mobilisation by mb, but also SRFA under certain conditions (Figure 2b), may both contribute to an increased Cu bioavailability to methanotrophs in the presence of Cu_x_S NPs.

Dissolved Cu concentrations in Cu_x_S NP suspensions with O_2_ varied among the pH values tested (Figure S3). Thermodynamic calculations predict that the limited dissolution of Cu_x_S NPs in absence of mb at pH 7.5 and at pH 8.5 (Figure S3b), in presence of O_2_ is due to the formation of Cu(hydr)oxide phases (Table S3). While Cu_x_S NPs are predicted to dissolve fully at pH 5 and 6 (Table S3), in absence of mb dissolution was slow under low O_2_ conditions and dissolved Cu concentrations were <0.8 μM until 8 h (Figure S3b).

At all pH values tested, all of the added mb is predicted to be present as Cu‐mb complexes under oxic conditions (Table S3), suggesting that mb can effectively compete with NOM constituents, like the fulvic acids used in our experiments, to bind Cu. Previous work by Pesch et al. (2013) demonstrated that mb can compete for Cu in the presence of humic acids through fast ligand exchange reactions, even when the binding capacity of humic acids exceeded that of mb (Pesch et al., 2013). At pH 5 and 6, Cu_x_S NPs are predicted to dissolve completely. A higher concentration of Cu is predicted to be complexed by mb than the added mb concentration (Table S3) due to mb's capability to complex two Cu atoms under high dissolved Cu concentrations (Pesch et al., 2012). Over the examined time period, dissolved Cu concentrations mobilised in NP suspensions differ when compared to the predicted dissolved Cu concentrations, probably because dissolved Cu concentrations have not reached equilibrium yet (Figure S3b), though equilibrium conditions are not a prerequisite for Cu acquisition. Adsorption processes could also play a role. At pH 5, for example, dissolved Cu concentrations did not increase under anoxic conditions in the presence of mb, possibly for a couple of reasons (Figure S3a). Firstly, the efficacy for Cu‐mb complex formation is pH‐dependent and mb binds more Cu with increasing pH, although the effect should be rather weak between pH 5 and 8 (Pesch et al., 2012). Secondly, changes in the surface charge of the NPs and the overall charge of the Cu‐free mb ligand as a function of pH may result in repulsive forces between Cu_x_S NPs and Cu‐free mb, thus limiting Cu_x_S NP dissolution. Several studies show that the point of zero charge for Cu sulphides lies between pH 1–4 (Bebie et al., 1998; Liu & Huang, 1992; Yin et al., 2019). Therefore, NPs are expected to be negatively charged under all pH conditions of this study, which would diminish the adsorption of mb due to electrostatic repulsion. A reduction in the surface charge could also lead to coagulation of Cu_x_S NPs, effectively reducing the surface area from which mb is able to mobilise Cu. Our results show that the effectiveness of mb on Cu_x_S NP dissolution is pH‐dependent and mb is most effective for Cu mobilisation from these Cu sulphides between pH 6–8.5.

### 4.2 Effects of pH on well‐crystalline Cu sulphide solubility

Well‐crystalline Cu sulphide solubility was strongly influenced by pH. Experimentally, dissolved Cu concentrations in CuS suspensions in absence of mb decreased with increasing pH between pH 6–8 (Figure 4). Higher dissolved Cu concentrations than would be expected in equilibrium with crystalline CuS were observed between pH 6–7. This could be due to partial dissolution of (less crystalline) CuS domains with a higher solubility that were not removed in the washing step following mineral synthesis. Ma et al. (2014) also observed dissolved Cu concentrations in excess of those predicted for crystalline CuS during Cu sulphide nanoparticle dissolution (Ma et al., 2014). They ascribed this to the formation of CuS nanoclusters that passed through filters thus contributing to the dissolved fraction. While CuFeS_2_ exhibited low Cu dissolution across the entire pH range investigated, both minerals exhibited low solubility above pH 7 under anoxic conditions (Figure 4), much like Cu_x_S NPs. While the solubility of Cu sulphides differs between mineral phases, limited dissolution above pH 7 under anoxic conditions appears to be ubiquitous across sulphide minerals investigated in this study.

The presence of mb seems to increase the solubility of Cu sulphide minerals, and increase total dissolved Cu concentrations, but the effectiveness of mb in this regard is, again, pH‐dependent. In CuFeS_2_ suspensions, mb increased dissolved Cu concentrations the most between pH 6–7 where the solubility of CuFeS_2_ was low and dissolved Cu concentrations did not exceed 0.9 μM (Figure 4). Dissolved Cu concentrations were particularly low above pH 7 in CuS suspensions in absence of mb. Incidentally, this is also where mb had the greatest effect on Cu mobilisation from CuS (Figure 4). Methanobactin increased dissolved Cu concentrations in the presence of Cu sulphide phases that have low solubility, particularly at pH values where Cu sulphide dissolution is otherwise inhibited. This observation supports the notion that the exudation of mb by methanotrophs is necessary at circumneutral pH to increase the kinetics of Cu sulphide dissolution.

In our dissolution experiments with CuFeS_2_ in absence of mb, dissolved Fe concentrations (Figure 6) were higher than dissolved Cu concentrations (Figure 4), whereas stoichiometric CuFeS_2_ dissolution would lead to equal concentrations. As previously mentioned, XRD analysis indicated that our natural CuFeS_2_ sample consisted of ~7% chamosite, an Fe(II) silicate mineral. Final dissolved Fe concentrations in CuFeS_2_ suspensions were compared with predicted Fe concentrations in equilibrium with chamosite (Figure S7). Predicted dissolved Fe concentrations in equilibrium with chamosite are close to those observed in dissolution experiments. Therefore, dissolved Fe concentrations in CuFeS_2_ suspensions under anoxic conditions possibly result, to a large extent, from chamosite dissolution. Metal ions other than Cu, such as Fe(II), likely compete for the complexation of mb. It is important, therefore, to consider whether the presence of dissolved Fe competes with Cu for mb, reducing the amount of mb available to form complexes with Cu.

Cu is complexed to mb in a tetrahedral geometry via two nitrogen (N) and two S moieties (Kim et al., 2004), functional groups ideal for binding soft and borderline metal ions like Cu(I) and Cu(II), as described by the hard and soft acid and base (HSAB) theory (see, e.g. Kraemer et al., 2015). Biogenic ligands are rarely specific to one metal (Kraemer et al., 2015; Schenkeveld, Oburger, et al., 2014; Schenkeveld, Schindlegger, et al., 2014) and mb is no exception in this regard (Choi, Do, et al., 2006; McCabe et al., 2017). Previous work suggested that mb can form complexes with Fe(III) (Choi, Do, et al., 2006). But, HSAB theory predicts that mb is more likely to form complexes with Fe(II), which, based on its charge‐to‐radius ratio, is considered a borderline Lewis acid according to HSAB theory (Pearson, 1963). There are multiple other functional groups in mb, like phenolates and carboxylates, which could also be involved in binding Fe(II) (Behling et al., 2008; El Ghazouani et al., 2011; Kim et al., 2004). To investigate whether mb interacts with Fe under our experimental conditions the UV–vis absorption spectra of mb were investigated in the presence of Fe(II) and Fe(III) (Figure S8 and S9, respectively). Changes in the spectra were observed upon the addition of Fe(II) only (Figure S8) indicating interactions between Fe(II) and mb.

Under anoxic conditions, interactions between Fe and mb may be the cause of increased Fe mobilisation at pH 7.5 and pH 8 (Figure 6). The effect of mb on dissolved Fe concentrations in the presence of O_2_ (Figure 8 and S6), which is discussed later, is even larger. In the context of Fe nutrition, competition between Fe and Cu for complexation by biogenic and synthetic chelating ligands has been extensively studied (Schenkeveld et al., 2012; Schenkeveld et al., 2015; Schenkeveld, Oburger, et al., 2014; Schenkeveld, Schindlegger, et al., 2014). Fe limitation, however, typically occurs under oxic conditions, in which Fe(III) is the dominant redox state; Fe(III) displacement from the chelate complex by Cu can compromise the effectiveness of Fe acquisition strategies. Despite possible interactions between Fe(II) and mb, dissolved Cu concentrations increased in the presence of mb (Figure 4), even when dissolved Fe concentrations were increasing (Figure 6). Our results show that Cu mobilisation by mb is still efficient even in the presence of potentially competing metals like Fe (Figure 4).

### 4.3 Cu (hydr)oxide formation and Cu availability

Following complete oxidation of Cu sulphides, Cu^2+^ ions are released into solution (Equation 1), which may form Cu (hydr)oxide phases depending on the solution pH. Aside from Cu (hydr)oxide phases (Todd & Sherman, 2003), oxidation of Cu sulphides may result in products with intermediate oxidation states like S^0^ and hydrophobic polysulphides (Acres et al., 2010; Li et al., 2017), and Fe (hydr)oxides in the case of CuFeS_2_. Cu (hydr)oxides are least soluble between pH 8.5–11 (Cuppett et al., 2006). This is consistent with thermodynamic calculations predicting decreasing dissolved Cu concentrations in equilibrium with Cu (hydr)oxides with increasing pH, at least over the examined pH range. Dissolved Cu concentrations in CuS and CuFeS_2_ suspensions under oxic conditions were compared with the predicted concentrations in equilibrium with Cu (hydr)oxide phases, in the presence and absence of mb, and as a function of pH (Figure 3). The difference between experimental dissolved Cu concentrations and model predictions at lower pH values (Figure 3) could be a result of the rather short reaction time (8 h), i.e. equilibrium conditions were not reached because of the slow oxidation kinetics of Cu sulphide minerals. Above pH 7, low dissolved Cu concentrations in absence of mb (Figure 3) may be due to precipitation of a secondary Cu (hydr)oxide phase passivating the mineral surface (Figure 3). Thermodynamic calculations corroborate the experimental results, predicting that dissolved Cu concentrations will increase in the presence of mb (Figure 3). The model predicts higher dissolved Cu concentrations than those observed experimentally suggesting that the experimental system has not reached equilibrium conditions.

The addition of mb to Cu sulphide suspensions increased dissolved Cu concentrations under pH conditions where Cu (hydr)oxides form (Figure 3). Yet, even without mb, the growth of MOB may be possible under these conditions. More precisely, predicted dissolved Cu concentrations in equilibrium with Cu (hydr)oxides are not lower than ~0.1 μM over the examined pH range (Figure 3), and based on previous laboratory investigations, growth of *M*. *trichosporium* OB3b cells can happen in cultures containing only 30 nM dissolved Cu (Semrau et al., 2010). Under these low Cu concentrations, however, the bacteria ceased to express pMMO, and concomitantly increased their expression of the Fe‐bearing sMMO. The Cu‐bearing pMMO was only expressed again when dissolved Cu concentrations in the growth medium reached at least 0.7 μM (Semrau et al., 2010). Therefore, low available Cu concentrations could reduce the efficacy of aerobic CH_4_ oxidation by inhibiting the expression of pMMO. Subsequently, the exudation of mb may be necessary to increase dissolved Cu concentrations where Cu sulphide oxidation is slow and to enhance the solubility of Cu (hydr)oxide phases. The efficacy of Cu mobilisation by mb from CuS and CuFeS_2_ in the presence of O_2_ is, however, pH‐dependent. Our results show that mb enhanced Cu mobilisation most effectively between pH 7–8.5 where dissolved Cu concentrations are limited in oxic systems.

In absence of mb, dissolved Fe concentrations were low under oxic conditions, which is most likely due to Fe (hydr)oxide formation (Figures 8 and S6). Upon addition of mb, dissolved Fe concentrations increased (Figures 8 and S6). As previously discussed, changes in the UV–vis absorption spectra of mb in the presence of Fe(II) indicate an interaction between Fe(II) and mb. It is difficult to discern from our experimental data whether increased dissolved Fe concentrations inhibit Cu mobilisation by mb. Under anoxic conditions mb had little effect on mobilised Fe concentrations and the sum of dissolved Cu and Fe mobilised from CuFeS_2_ did not exceed the added mb concentration. Under oxic conditions the sum of final dissolved Cu and Fe concentrations mobilised from CuFeS_2_ did exceed the added mb concentration, but the sum of the difference in final Fe and Cu concentrations between the mb treatment and the control, respectively, was less than the added mb concentration (20 μM). At pH 6, in the presence of O_2_, final dissolved Fe concentrations increased considerably and accounted for ~3/4 of the added mb concentration (Figure 8); however, the effect of mb on dissolved Cu concentrations mobilised from CuFeS_2_ was negligible (Figure 7). While it is possible that interactions between mb and Fe could inhibit Cu mobilisation from CuFeS_2_ by mb, the sum of the difference in Fe and Cu concentrations (Figures 7 and 8) between treatments was only ~15 μM, indicating that some of the added mb may still be present as free mb. Despite increased dissolved Fe concentrations in the presence of mb and O_2_, dissolved Cu concentrations mobilised from CuFeS_2_ also increased between pH 6.5–8.5. Still, exudation of mb in oxic environments, where CuFeS_2_ is the dominant Cu source, may not be a prerequisite for Cu acquisition under slightly acidic pH conditions where dissolved Cu concentrations are high irrespective of mb.

## 5 CONCLUSIONS

By combining dissolution experiments with chemical equilibrium modelling, we have shown that mb is effectively increasing the dissolved Cu concentrations in the presence of a variety of Cu sulphide phases that may potentially limit Cu bioavailability in environmental systems over time scales relevant for methanotrophic chalkophore exudation and subsequent Cu‐uptake. In dissolution experiments, the reactivity of mb towards Cu differed, depending on the Cu sulphide mineral phase tested. However, a reconciling observation for all Cu sulphide mineral phases was that mb consistently promoted Cu mobilisation between pH 7.5–8.5, also under oxic conditions where Cu (hydr)oxides can precipitate as a result of Cu sulphide oxidation (and can thus constrain Cu bioavailability). The presence of mb enhances dissolved Cu concentrations most significantly for nanoparticulate Cu sulphide phases, which, in the absence of mb, exhibit high stability, even in the presence of O_2_. Using thermodynamic calculations, we have demonstrated which secondary mineral phases may constrain dissolved Cu concentrations following sulphide oxidation and quantified the speciation of Cu in the presence of mb, sulphides and SRFA. As Cu is an essential nutrient for several microbes, the implications of mb overcoming geochemical constraints to microbial Cu acquisition goes beyond a better understanding of Cu acquisition by methanotrophs. The effects of Cu limitation to growth have been observed in several microbial species: from the terrestrial archaeon *Nitrosospheara viennensis* (Reyes, Hodgskiss, Baars, et al., 2020), to the marine diatom *Thalassiosira oceanica* (Sunda & Huntsman, 1995). While there are multiple cell‐associated mechanisms through which microbes acquire Cu (Reyes, Hodgskiss, Kerou, et al., 2020), the exudation of ligands such as mb allows methanotrophs to increase Cu availability amidst an array of geochemical constraints. Additionally, mb may provide methanotrophs with a competitive edge in the presence of microbes that cannot exude chalkophores and/or are lacking the relevant membrane transporters. Our experimental results clearly demonstrate that mb mobilises Cu in the presence of sulphide, providing a pathway for methanotrophs to acquire Cu for enhanced methane oxidation resulting in reduced emissions and mitigating its impact on global warming. It remains unclear, whether the capacity of methanotrophs to produce mb and acquire Cu was possibly developed, driven by evolutionary pressure, as a biochemical strategy to overcome Cu limitation in oxic‐anoxic transitions zones of lakes, for example, where Cu is generally limited, and competition for CH_4_ between MOx and anaerobic methane oxidation is most likely to occur. Obviously, the efficiency of Cu acquisition has important implications for the mitigation of methane emissions from aquatic systems.

## Supporting information


Appendix S1

## Data Availability

The data that support the findings of this study are available in the supplementary material of this article.
