# Supplementary material for: Copper mobilisation from Cu sulphide minerals by methanobactin: Effect of pH, oxygen and natural organic matter
Source: Geobiology. 2022 Jun 18;20(5):690–706. doi: 10.1111/gbi.12505 (PMC9544142; doi:10.1111/gbi.12505)
Supplement: Supplementary file 1 — Appendix S1 [file GBI-20-690-s001.docx]

**Supporting Information**

**Copper mobilisation from (Cu-)sulphide minerals by methanobactin: effect of pH, oxygen and natural organic matter**

Danielle D. Rushworth^1^, Iso Christl^2^, Naresh Kumar^3^, Kevin Hoffmann^2^, Ruben Kretzschmar^2^, Moritz F. Lehmann^4^, Walter D. C. Schenkeveld^3^, Stephan M. Kraemer^1^

^1^Centre for Microbiology and Environmental Systems Science, University of Vienna, Vienna, Austria (stephan.kraemer@univie.ac.at)

^2^Soil Chemistry, Institute of Biogeochemistry and Pollutant Dynamics, ETH Zurich, Switzerland

^3^Soil Chemistry and Chemical Soil Quality, Environmental Sciences, Wageningen University, Wageningen, Netherlands (walter.schenkeveld@wur.nl)

^4^Dept. of Environmental Geosciences, University of Basel, Basel, Switzerland

**Content**

**Figures pg.**

**S1** XRD analysis of synthetic covellite and natural chalcopyrite **2**

**S2** Cu mobilisation by methanobactin from Cu_x_S nanoparticles (pH 7.5) under 1 % oxygen **3**

**S3** Cu mobilisation by methanobactin from Cu_x_S nanoparticles at pH 5, pH 6 and pH 8.5 under anoxic conditions and 1 % oxygen **4**

**S4** Cu mobilisation by methanobactin from covellite at pH 6.5, pH 7, pH 8 and pH 8.5 under anoxic conditions and under 21 % oxygen  **5**

**S5** Cu mobilisation by methanobactin from chalcopyrite at pH 6.5, pH 7, pH 8 and pH 8.5 under anoxic conditions and under 21 % oxygen  **6**

**S6** Fe mobilisation by methanobactin from chalcopyrite at pH 6.5, pH 7, pH 8 and pH 8.5 under anoxic conditions and under 21 % oxygen  **7**

**S7** Fe mobilisation from natural chalcopyrite dissolution experiments compared to the predicted solubility of chamosite as a function of pH  **8**

**S8** UV-vis absorption spectra for methanobactin (pH 7.5) in the presence of varied Fe (II) concentrations  **9**

**S9** UV-vis absorption spectra for methanobactin (pH 7.5) in the presence of varied Fe (III) concentrations **10**

**Tables**

**S1** Final experimental solution concentrations and predicted S speciation for Cu_x_S NP suspensions **11**

**S2** Generic parameters used in the NICA-Donnan model to describe the binding of H^+^ and Cu^2+^ to fulvic acids **11**

**S3** Speciation predictions for Cu_x_S NP suspensions under oxic conditions **12**

**S4** Input data for thermodynamic modelling of bulk phase minerals **13**

**S5** Results of statistical analysis of the effect of mb on dissolved Fe concentrations under anoxic conditions **14**

**Tabulated data for all Figures 15**

(a) – Covellite

(b) – Chalcopyrite


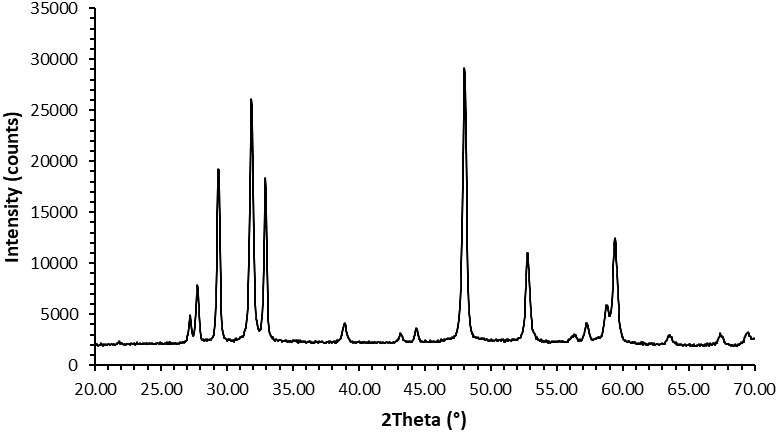


**FIGURE S1** X-ray diffractogram of (a) pure phase synthetic covellite and (b) natural chalcopyrite (red line shows the peak signal for pure chalcopyrite).


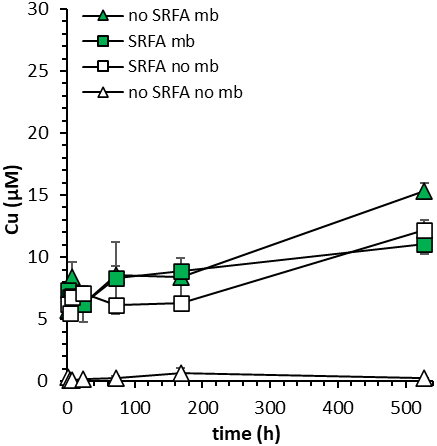

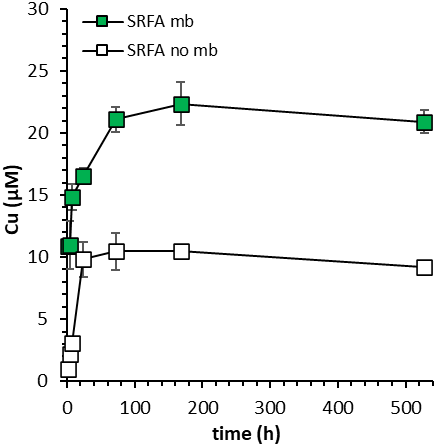


(a) – pH 7.5 NPs with and without SRFA - 1% oxygen

(b) – pH 7.5 NPs – with SRFA only - 21% oxygen

**FIGURE S2** Cu mobilisation from Cu_x_S NPs (10 mM NaCl) in the presence of Suwannee River fulvic acids (SRFA) (squares; 5 mg C L^-1^) and absence of SRFA (triangles) at pH 7.5 under (a) low oxygen conditions (pO_2_ = 0.01 atm) and under high oxygen conditions (pO_2_ = 0.21 atm). Filled symbols show mb treatment (19 µM mb) and open symbols show controls (no mb). Error bars represent the range of duplicate measurements.


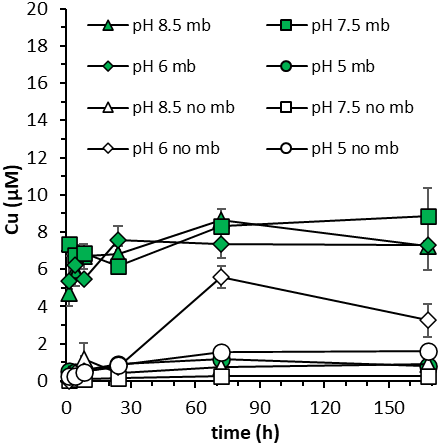


(a) – NPs with SRFA - anoxic

(b) – NPs with SRFA - 1% oxygen


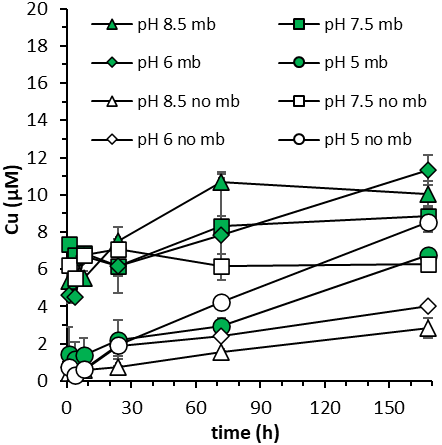


**FIGURE S3** Cu mobilisation from Cu_x_S NPs in the presence of 5 mg C L^-1^ Suwannee River fulvic acids (SRFA) at pH 5 (circles), pH 6.5 (diamonds), pH 7.5 (squares) and pH 8.5 (triangles) under (a) anoxic conditions and (b) low oxygen conditions (pO_2_ = 0.01 atm). Filled symbols show mb treatments (19 µM mb) and open symbols show controls (no mb). Error bars represent the range of duplicate measurements.


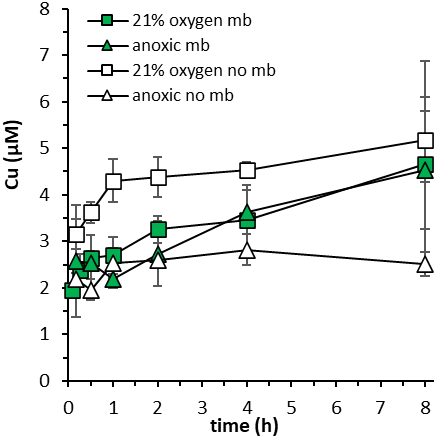

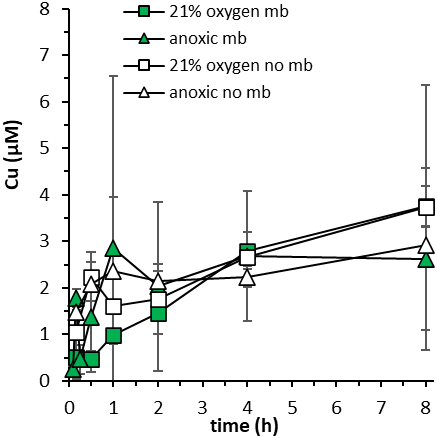

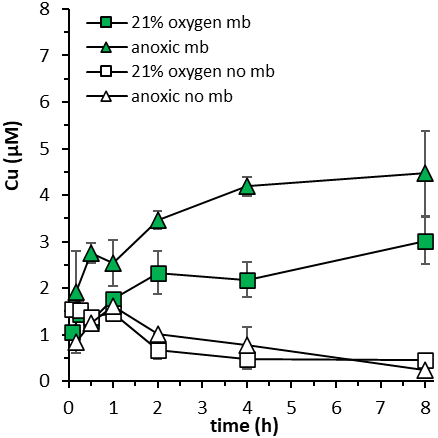


(a) –pH 6.5 CuS

(b) – pH 7 CuS

(c) – pH 8 CuS

(d) – pH 8.5 CuS


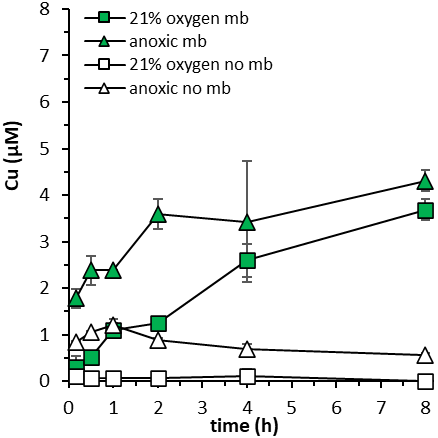


**FIGURE S4** Cu mobilisation from covellite (2 g L^-1^, 10 mM NaCl) under oxic conditions (squares; pO_2_ = 0.21 atm) and under anoxic conditions (triangles) at (a) pH 6.5 (b) pH 7 (c) pH 8 and (d) pH 8.5. Filled symbols show mb treatments (20 µM mb) and open symbols show controls (no mb). Error bars represent the range of duplicate measurements.


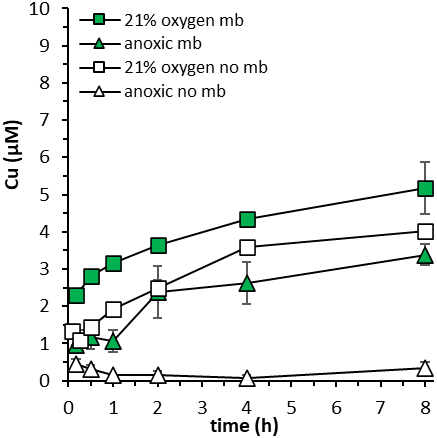

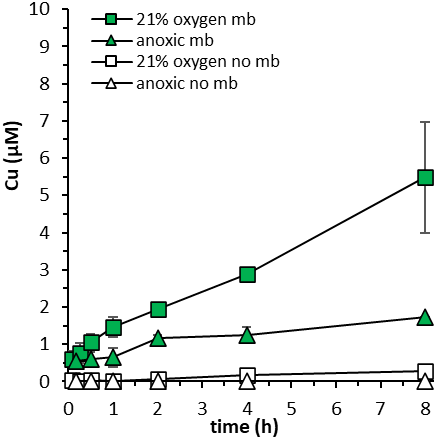

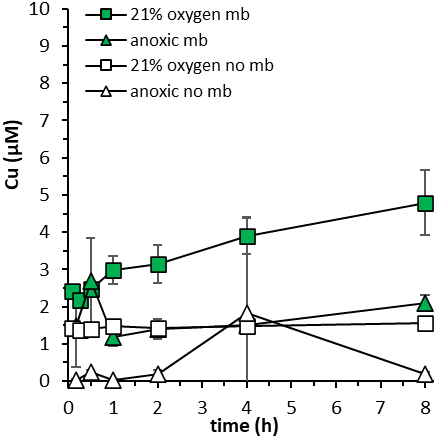


(a) – pH 6.5 CuFeS_2_

(b) – pH 7 CuFeS_2_

(c) – pH 8 CuFeS_2_

(d) – pH 8.5 CuFeS_2_


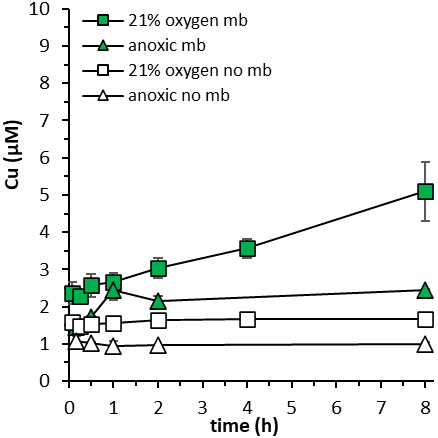


**FIGURE S5** Cu mobilisation from chalcopyrite (2 g L^-1^, 10 mM NaCl) under oxic conditions (squares; pO_2_ = 0.21 atm) and under anoxic conditions (triangles) at (a) pH 6.5 (b) pH 7 (c) pH 8 and (d) pH 8.5. Filled symbols show mb treatments (20 µM mb) and open symbols show controls (no mb). Error bars represent the range of duplicate measurements.


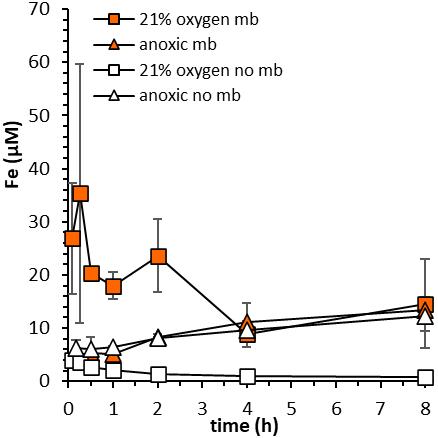

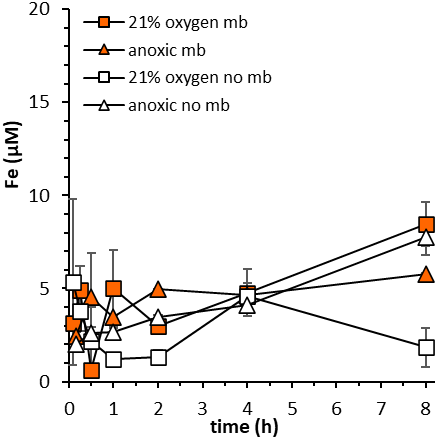

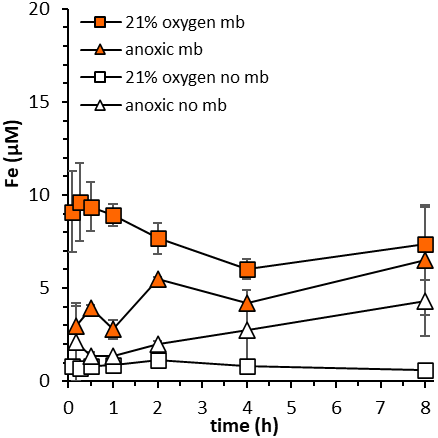

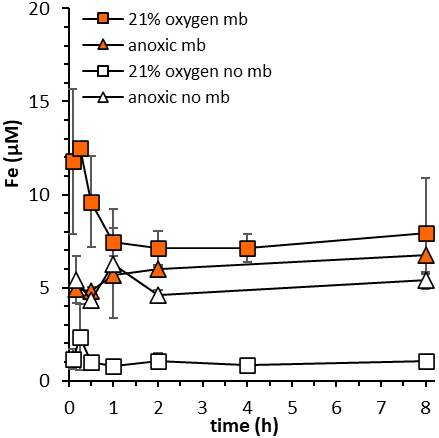


(a) - pH 6.5 CuFeS_2_

(b) – pH 7 CuFeS_2_

(c) – pH 8 CuFeS_2_

(d) – pH 8.5 CuFeS_2_

**FIGURE S6** Fe mobilisation from chalcopyrite (2 g L^-1^, 10 mM NaCl) under oxic conditions (squares; pO_2_ = 0.21 atm) and under anoxic conditions (triangles) at (a) pH 6.5 (b) pH 7 (c) pH 8 and (d) pH 8.5. Filled symbols show mb treatments (20 µM mb) and open symbols show controls (no mb). Error bars represent the range of duplicate measurements. Note the different scale for pH 6.5.

**FIGURE S7** Fe mobilisation from chalcopyrite under anoxic conditions (2 g L^-1^, 10 mM NaCl) at 8 hours in the absence of mb as a function of pH (orange circles). Error bars represent the range of duplicate measurements. The dotted line represents the predicted solubility of Fe in equilibrium with chamosite under anoxic conditions as a function of pH calculated using PHREEQC (10 mM NaCl, pe -2.5).


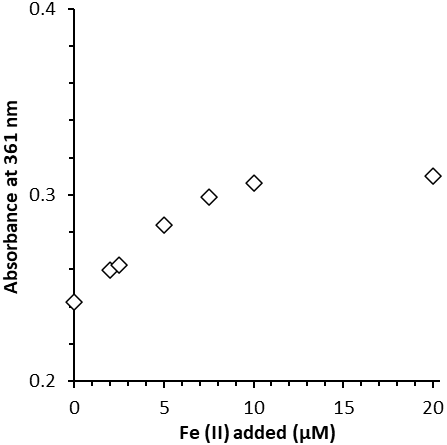


y = 0.0076x + 0.2438

R^2^ = 0.9947

(a)

(b)


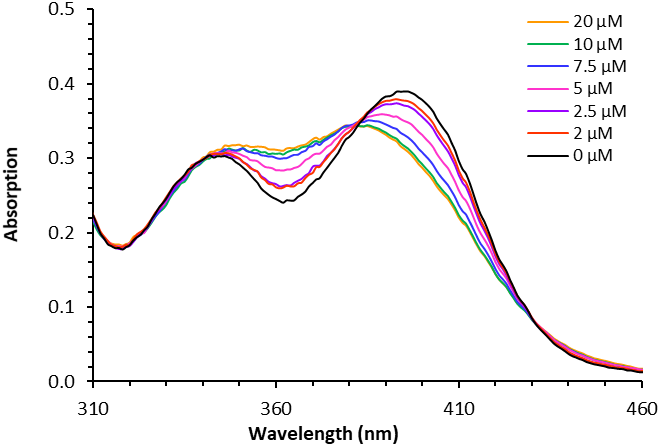


**FIGURE S8** (a) UV-vis absorption spectra of 20 µM mb in the presence of varying Fe(II) concentrations up to a 1:1 molar ratio and (pH 7.5, 10 mM NaCl) prepared under anoxic conditions. The arrow indicates the direction of spectral change at 361 nm with increasing Fe (II). (b) UV-vis absorption 20 µM mb at 361 nm as a function of Fe(II) (pH 7.5, 10 mM NaCl).

**FIGURE S9** UV-vis absorption spectra of 26 µM mb in presence of varying Fe(III) concentrations (pH 7.5, 10 mM NaCl) prepared under anoxic conditions.

**Equilibrium calculations for Cu_x_S NP suspensions**

Equilibrium predictions for the Cu speciation in the experimental Cu_x_S NP suspensions were made at 25 °C (experimental temperature) in Visual MINTEQ. Recent work by Hoffmann et al. (2020) demonstrated that covellite was the dominant phase in Cu_x_S NP suspensions. Therefore, solid Cu was incorporated to the model as CuS. The solids concentration was selected based on the final Cu concentrations in dissolution experiments (Table S1). In the model, half of the total dissolved sulphide concentration added to synthesise Cu_x_S NPs was accounted for by solid phase CuS, the speciation of the rest was determined using the Henry’s constant for hydrogen sulphide and the calculated dissolved portion was added as dissolved sulphide (Table S1). Na^+^ and Cl^-^ were added to the model solution phases and the predicted ionic strength was equal to the experimental NaCl concentration ± 1. Solution pH was fixed by adding dissolved H^+^ at the relevant pH concentration. The redox couples Cu^+^/Cu^2+^ and HS^-^/SO_4_^2-^ were defined in the input file for all calculations. Cu binding to SRFA was described using the Non-Ideal consistent Competitive Adsorption (NICA)-Donnan model using the so-called “generic” parameters for H^+^ and Cu^2+^ binding to fulvic acids (FA) (Kinniburgh et al., 1996; Koopal, Saito, Pinheiro, & Riemsdijk, 2005; Milne, Kinniburgh, van Riemsdijk, & Tipping, 2003) (Table S2). 100 % of the added FA was assumed to be active dissolved organic matter (DOM). An active DOM to dissolved organic carbon (DOC) ratio of 1.9 was used, corresponding to the elemental composition of the SRFA standard II. Equilibrium predictions were also made for Cu_x_S NP suspensions under oxic conditions. O_2_ was added as a gas and redox potential (pe) was not fixed under oxic conditions. The concentrations of all solutes were adjusted to account for volume changes to NP suspensions during pH adjustment (Table SI).

**TABLE S1** (a) Final reactant concentrations in Cu_x_S nanoparticle suspensions during both oxic and anoxic dissolution experiments. (b) Distribution of dissolved sulphur species at each pH.

| (a) Final reactant concentrations | | | | | | | |
| --- | --- | --- | --- | --- | --- | --- | --- |
|  | **CuCl_2_ (µM)** | **Na_2_S (µM)** | **NaCl (mM)** | **SRFA**  **(mg C L^-1^)** | **MOPS (mM)** | **MES (mM)** | **DEPP (mM)** |
| **pH 5 SRFA** | 47.4 | 94.7 | 9.47 | 4.74 | 0.95 | - | 2.16 |
| **pH 6 SRFA** | 45.5 | 91.0 | 9.10 | 4.55 | 0.91 | 40.93 | - |
| **pH 7.5 SRFA** | 47.5 | 95.0 | 10.00 | 4.75 | 0.95 | - | - |
| **pH 7.5 no SRFA** | 47.5 | 95.0 | 10.00 | - | 0.95 | - | - |
| **pH 8.5 SRFA** | 46.7 | 93.4 | 9.34 | 4.67 | 0.93 | - | 16.60 |
|  | (b) S speciation (%) | | | (b) S speciation (µM) | | | |
|  | **H_2_S _(g)_** | **H_2_S _(aq)_** | **HS^-^ _(aq)_** | **H_2_S _(g)_** | **H_2_S _(aq)_** | **HS^-^ _(aq)_** | **Total S _(aq)_** |
| **pH 5** | 39.8 | 59.6 | 0.6 | 18.9 | 28.2 | 0.3 | 28.5 |
| **pH 6** | 37.9 | 56.7 | 5.4 | 17.2 | 25.8 | 2.5 | 28.3 |
| **pH 7.5** | 14.2 | 21.3 | 64.4 | 6.8 | 10.1 | 30.6 | 40.8 |
| **pH 8.5** | 2.1 | 3.1 | 94.8 | 1.0 | 1.5 | 44.3 | 45.7 |

Note: Reactant concentrations in each pH treatment differ to that of the original suspensions prepared at pH 7.5 due to dilution of nanoparticle suspensions following pH adjustment. Sulphur distributions assume that 50% of added sulphide is bound to Cu in the solid phase. SRFA = Suwannee River fulvic acids. MOPS, MES and DEPP are pH buffers.

**TABLE S2** Generic parameters used in the Non-Ideal consistent Competitive Adsorption (NICA)-Donnan model to describe the binding of H^+^ and Cu^2+^ to fulvic acids (FA) taken from Milne et al. 2003.

| **Parameter** | **Generic FA Parameters**  **(Milne et al., 2003)** |
| --- | --- |
| b(Vd) | 0.57 |
|  |  |
| Qmax1 | 5.88 |
| p1 | 0.59 |
| log KH1 | 2.40 |
| nH1 | 0.66 |
| log KCu1 | 0.26 |
| nCu1 | 0.53 |
|  |  |
| Qmax2 | 1.86 |
| p2 | 0.70 |
| log KH2 | 8.60 |
| nH2 | 0.76 |
| log KCu2 | 8.26 |
| nCu2 | 0.36 |

*Note:* FA denotes fulvic acids; CuS is solid covellite; S(-II) is dissolved sulphide; pe is the negative logarithm of the electron concentration in solution; mb is methanobactin; Total Cu_diss_ is the sum of dissolved Cu species; Total Cu-mb is the sum of Cu-mb species; Total Cu-FA is the Cu concentration bound to FA and Free mb is the total mb concentration not complexed to Cu.

**TABLE S3** Equilibrium predictions for Cu speciation in the Cu_x_S NP suspensions at pH 5, 6, 7.5 and 8.5 in presence and absence of mb, at levels of O_2_ in equilibrium with the atmosphere.

| **Input parameters** | | | | |  | | **Solid and dissolved Cu speciation** | | | | | | | |
| --- | --- | --- | --- | --- | --- | --- | --- | --- | --- | --- | --- | --- | --- | --- |
| pH | FA  (mg C L^-1^) | CuS (µM) | S(-II)  (µM) | mb  (µM) | | Equilibrium  phase | | Equilibrium phase Cu (M) | Total Cu_diss_ (M) | Total Cu-mb (M) | Total Cu-FA (M) | Total  Cu^*^  (M) | Free mb  (M) |  |
| 7.5 | 4.75 | 47.5 | 40.8 | 0 | | Cu(OH)_2_(s) | | 2.7E-05 | 2.1E-05 | 0 | 1.5E-05 | 0.6E-05 | 0 |  |
| 7.5 | 4.75 | 47.5 | 40.8 | 0 | | Tenorite(c) | | 4.2E-05 | 5.8E-06 | 0 | 5.7E-06 | 0.1E-05 | 0 |  |
| 7.5 | 4.75 | 47.5 | 40.8 | 19 | | Cu(OH)_2_(s) | | 0 | 4.8E-05 | 1.9E-05 | 1.4E-05 | 1.5E-05 | 2.0E-12 |  |
| 7.5 | 4.75 | 47.5 | 40.8 | 19 | | Tenorite(c) | | 2.2E-05 | 2.6E-05 | 1.9E-05 | 5.7E-06 | 0.1E-05 | 1.4E-10 |  |
| 5 | 4.74 | 47.4 | 28.5 | 0 | | Cu(OH)_2_(s) | | 0 | 4.7E-05 | 0 | 1.3E-05 | 3.4E-05 | 0 |  |
| 5 | 4.74 | 47.4 | 28.5 | 0 | | Tenorite(c) | | 0 | 4.7E-05 | 0 | 1.3E-05 | 3.4E-05 | 0 |  |
| 5 | 4.74 | 47.4 | 28.5 | 19 | | Cu(OH)_2_(s) | | 0 | 4.7E-05 | 2.6E-05 | 9.2E-06 | 1.1E-05 | 1.4E-09 |  |
| 5 | 4.74 | 47.4 | 28.5 | 19 | | Tenorite(c) | | 0 | 4.7E-05 | 2.6E-05 | 9.2E-06 | 1.1E-05 | 1.4E-09 |  |
| 6 | 4.55 | 45.5 | 28.3 | 0 | | Cu(OH)_2_(s) | | 0 | 4.5E-05 | 0 | 1.6E-05 | 2.9E-05 | 0 |  |
| 6 | 4.55 | 45.5 | 28.3 | 0 | | Tenorite(c) | | 0 | 4.5E-05 | 0 | 1.6E-05 | 2.9E-05 | 0 |  |
| 6 | 4.55 | 45.5 | 28.3 | 19 | | Cu(OH)_2_(s) | | 0 | 4.5E-05 | 2.3E-05 | 1.0E-05 | 1.2E-05 | 1.5E-10 |  |
| 6 | 4.55 | 45.5 | 28.3 | 19 | | Tenorite(c) | | 0 | 4.5E-05 | 2.3E-05 | 1.1E-05 | 1.1E-05 | 1.5E-10 |  |
| 8.5 | 4.67 | 46.7 | 45.7 | 0 | | Cu(OH)_2_(s) | | 3.9E-05 | 8.0E-06 | 0 | 7.6E-06 | 3.8E-07 | 0 |  |
| 8.5 | 4.67 | 46.7 | 45.7 | 0 | | Tenorite(c) | | 4.4E-05 | 2.7E-06 | 0 | 2.7E-06 | 8.5E-09 | 0 |  |
| 8.5 | 4.67 | 46.7 | 45.7 | 19 | | Cu(OH)_2_(s) | | 1.9E-05 | 2.7E-05 | 1.9E-05 | 7.6E-06 | 3.8E-07 | 4.1E-12 |  |
| 8.5 | 4.67 | 46.7 | 45.7 | 19 | | Tenorite(c) | | 2.5E-05 | 2.2E-05 | 1.9E-05 | 2.7E-06 | 8.1E-09 | 1.9E-10 |  |

**Thermodynamic modelling for bulk phase minerals**

Equilibrium predictions made using the modelling programme PHREEQC were calculated at 21 °C (experimental temperature). The pH and pe were fixed by allowing the addition of NaOH and O_2_ (g), respectively.

**TABLE S4** - Input data for PHREEQC bulk phase mineral modelling, 20 °C.

| **equilibrium**  **phase** | **Ksp** | **Na^+^**  **(mmol L^-1^)** | **Cl^-^**  **(mmol L^-1^)** | **pe** | **log O_2_** | **log CO_2_** |
| --- | --- | --- | --- | --- | --- | --- |
| Cu(OH)_2_ | 8.67 | 10 | 10 | default | -0.68 | -3.5 |
| Tenorite (CuO) | 7.64 | 10 | 10 | default | -0.68 | -3.5 |

**TABLE S5** P-values of ANOVA statistical analysis on the effect of methanobactin on dissolved Fe concentrations in CuFeS_2_ dissolution experiments under anoxic conditions.

|  | **pH 6** | **pH 6.5** | **pH 7** | **pH 7.5** | **pH 8** | **pH 8.5** |
| --- | --- | --- | --- | --- | --- | --- |
| mb | 0.203 | 0.901 | 0.080 | 0.000 | 0.006 | 0.299 |

*Note:* Statistical analysis was conducted in Excel using the ANOVA: Two-factor with replication tool (α = 0.05).

**References**

Kinniburgh, D. G., Milne, C. J., Benedetti, M. F., Pinheiro, J. P., Filius, J., Koopal, L. K., & Van Riemsdijk, W. H. (1996). Metal Ion Binding by Humic Acid:  Application of the NICA-Donnan Model. *Environmental Science & Technology, 30*(5), 1687-1698. doi:10.1021/es950695h

Koopal, L. K., Saito, T., Pinheiro, J. P., & Riemsdijk, W. H. v. (2005). Ion binding to natural organic matter: General considerations and the NICA–Donnan model. *Colloids and Surfaces A: Physicochemical and Engineering Aspects, 265*(1), 40-54. doi:<https://doi.org/10.1016/j.colsurfa.2004.11.050>

Milne, C. J., Kinniburgh, D. G., van Riemsdijk, W. H., & Tipping, E. (2003). Generic NICA−Donnan Model Parameters for Metal-Ion Binding by Humic Substances. *Environmental Science & Technology, 37*(5), 958-971. doi:10.1021/es0258879

Data Table 1. Cu mobilised from Cu_x_S NPs (10 mM NaCl) in the presence of Suwannee River fulvic acids (SRFA) and absence of SRFA at pH 7.5 under (a) anoxic conditions and (b) under oxic conditions (pO_2_ = 0.21 atm) in the presence and absence of 19 µM mb. The range is the range of duplicate measurements. This table shows data from Figure 1.

Figure 1, Panel (a) Mobilised Cu concentrations under anoxic conditions (µM)

| time (h) | no SRFA  no mb | range | no SRFA mb | range | SRFA  no mb | range | SRFA  mb | range |
| --- | --- | --- | --- | --- | --- | --- | --- | --- |
| 1 | 0.0 | 0.0 | 6.9 | 0.1 | 0.0 | 0.0 | 7.3 | 0.0 |
| 4 | 0.1 | 0.0 | 6.9 | 0.7 | 0.1 | 0.0 | 6.7 | 0.4 |
| 8 | 0.1 | 0.0 | 7.6 | 0.7 | 0.1 | 0.0 | 6.9 | 0.0 |
| 24 | 0.1 | 0.0 | 7.3 | 0.4 | 0.2 | 0.0 | 6.2 | 0.0 |
| 72 | 0.2 | 0.0 | 8.2 | 1.0 | 0.2 | 0.0 | 8.3 | 0.4 |
| 168 | 0.3 | 0.2 | 9.2 | 2.6 | 0.3 | 0.1 | 8.9 | 1.5 |
| 528 | 1.0 | 0.0 | 12.0 | 2.9 | 1.1 | 0.0 | 11.1 | 1.5 |

Figure 1, Panel (b) Mobilised Cu concentrations under oxic conditions (µM)

| time (h) | SRFA  no mb | range | SRFA  mb | range |
| --- | --- | --- | --- | --- |
| 1 | 1.0 | 0.3 | 10.9 | 0.0 |
| 4 | 2.2 | 1.9 | 10.9 | 0.3 |
| 8 | 3.1 | 1.0 | 14.8 | 0.1 |
| 24 | 9.8 | 0.6 | 16.6 | 1.4 |
| 72 | 10.5 | 1.0 | 21.1 | 1.5 |
| 168 | 10.5 | 1.7 | 22.4 | 0.2 |
| 528 | 9.2 | 0.9 | 20.9 | 0.2 |

Data Table 2. Cu concentrations mobilised from well-crystalline (a) covellite and (b) chalcopyrite after 8 hours under oxic conditions in the presence and absence of 20 µM mb (2 g L^-1^ solids, 10 mM NaCl) and Cu concentrations in equilibrium with Cu(OH)_2_ and tenorite (CuO) with and without 20 µM mb calculated using PHREEQC (10 mM NaCl) all as a function of pH. The range is the range of duplicate measurements. Dashes denote no data. This table shows data from Figure 2.

Figure 2, Panel (a) Cu concentrations (µM) mobilised or in equilibrium with covellite

| pH | covellite no mb | range | covellite  mb | range | tenorite no mb | tenorite mb | Cu(OH)_2_  no mb | Cu(OH)_2_  mb |
| --- | --- | --- | --- | --- | --- | --- | --- | --- |
| 6 | 4.6 | 1.0 | 5.3 | 0.2 | 98.4 | 136.9 | 1039.0 | 1079.0 |
| 6.1 | - | - | - | - | 62.3 | 100.6 | 655.1 | 694.7 |
| 6.2 | - | - | - | - | 39.5 | 77.3 | 414.5 | 454.2 |
| 6.3 | - | - | - | - | 25.2 | 62.1 | 263.2 | 302.8 |
| 6.4 | - | - | - | - | 16.0 | 52.1 | 167.7 | 207.2 |
| 6.5 | 5.2 | 0.9 | 4.7 | 2.2 | 10.3 | 45.1 | 107.3 | 146.6 |
| 6.6 | - | - | - | - | 6.6 | 39.9 | 68.9 | 108.0 |
| 6.7 | - | - | - | - | 4.3 | 35.9 | 44.6 | 83.3 |
| 6.8 | - | - | - | - | 2.8 | 32.6 | 29.1 | 67.2 |
| 6.9 | - | - | - | - | 1.8 | 29.7 | 19.2 | 56.6 |
| 7 | 3.7 | 2.6 | 3.8 | 0.4 | 1.2 | 27.4 | 12.8 | 49.2 |
| 7.1 | - | - | - | - | 0.8 | 25.4 | 8.7 | 43.8 |
| 7.2 | - | - | - | - | 0.6 | 23.9 | 6.1 | 39.5 |
| 7.3 | - | - | - | - | 0.4 | 22.7 | 4.3 | 35.9 |
| 7.4 | - | - | - | - | 0.3 | 21.9 | 3.2 | 32.6 |
| 7.5 | 1.1 | 0.1 | 3.1 | 0.5 | 0.2 | 21.3 | 2.5 | 29.8 |
| 7.6 | - | - | - | - | 0.2 | 20.9 | 2.0 | 27.5 |
| 7.7 | - | - | - | - | 0.2 | 20.6 | 1.7 | 25.6 |
| 7.8 | - | - | - | - | 0.1 | 20.4 | 1.4 | 24.1 |
| 7.9 | - | - | - | - | 0.1 | 20.3 | 1.3 | 23.1 |
| 8 | 0.5 | 0.0 | 3.0 | 0.5 | 0.1 | 20.2 | 1.2 | 22.4 |
| 8.1 | - | - | - | - | 0.1 | 20.2 | 1.1 | 21.9 |
| 8.2 | - | - | - | - | 0.1 | 20.2 | 1.1 | 21.6 |
| 8.3 | - | - | - | - | 0.1 | 20.1 | 1.0 | 21.4 |
| 8.4 | - | - | - | - | 0.1 | 20.1 | 1.0 | 21.2 |
| 8.5 | 0.0 | 0.0 | 3.7 | 0.2 | 0.1 | 20.1 | 1.0 | 21.2 |

Figure 2, Panel (b) Cu concentrations (µM) mobilised or in equilibrium with chalcopyrite

| pH | chalcopyrite no mb | range | chalcopyrite mb | range | tenorite no mb | tenorite mb | Cu(OH)_2_  no mb | Cu(OH)_2_  mb |
| --- | --- | --- | --- | --- | --- | --- | --- | --- |
| 6 | 4.6 | 1.0 | 5.3 | 0.2 | 98.4 | 136.9 | 1039.0 | 1079.0 |
| 6.1 | - | - | - | - | 62.3 | 100.6 | 655.1 | 694.7 |
| 6.2 | - | - | - | - | 39.5 | 77.3 | 414.5 | 454.2 |
| 6.3 | - | - | - | - | 25.2 | 62.1 | 263.2 | 302.8 |
| 6.4 | - | - | - | - | 16.0 | 52.1 | 167.7 | 207.2 |
| 6.5 | 5.2 | 0.9 | 4.7 | 2.2 | 10.3 | 45.1 | 107.3 | 146.6 |
| 6.6 | - | - | - | - | 6.6 | 39.9 | 68.9 | 108.0 |
| 6.7 | - | - | - | - | 4.3 | 35.9 | 44.6 | 83.3 |
| 6.8 | - | - | - | - | 2.8 | 32.6 | 29.1 | 67.2 |
| 6.9 | - | - | - | - | 1.8 | 29.7 | 19.2 | 56.6 |
| 7 | 3.7 | 2.6 | 3.8 | 0.4 | 1.2 | 27.4 | 12.8 | 49.2 |
| 7.1 | - | - | - | - | 0.8 | 25.4 | 8.7 | 43.8 |
| 7.2 | - | - | - | - | 0.6 | 23.9 | 6.1 | 39.5 |
| 7.3 | - | - | - | - | 0.4 | 22.7 | 4.3 | 35.9 |
| 7.4 | - | - | - | - | 0.3 | 21.9 | 3.2 | 32.6 |
| 7.5 | 1.1 | 0.1 | 3.1 | 0.5 | 0.2 | 21.3 | 2.5 | 29.8 |
| 7.6 | - | - | - | - | 0.2 | 20.9 | 2.0 | 27.5 |
| 7.7 | - | - | - | - | 0.2 | 20.6 | 1.7 | 25.6 |
| 7.8 | - | - | - | - | 0.1 | 20.4 | 1.4 | 24.1 |
| 7.9 | - | - | - | - | 0.1 | 20.3 | 1.3 | 23.1 |
| 8 | 0.5 | 0.0 | 3.0 | 0.5 | 0.1 | 20.2 | 1.2 | 22.4 |
| 8.1 | - | - | - | - | 0.1 | 20.2 | 1.1 | 21.9 |
| 8.2 | - | - | - | - | 0.1 | 20.2 | 1.1 | 21.6 |
| 8.3 | - | - | - | - | 0.1 | 20.1 | 1.0 | 21.4 |
| 8.4 | - | - | - | - | 0.1 | 20.1 | 1.0 | 21.2 |
| 8.5 | 0.0 | 0.0 | 3.7 | 0.2 | 0.1 | 20.1 | 1.0 | 21.2 |

Data Table 3. Cu mobilised from covellite and chalcopyrite (both 2 g L^-1^, 10 mM NaCl) under anoxic conditions at (a) pH 6 (10 mM MES buffer), (b) 6.5 (10 mM MES buffer), (c) pH 7 (10 mM MOPS buffer), (d) pH 7.5 (10 mM MOPS buffer), (e) pH 8 (10 mM PIPPS buffer) and (f) pH 8.5 (10 mM PIPPS buffer) in the absence and presence of 20 µM mb. The range is the range of duplicate measurements (except at pH 7 CuS mb where the number in the range column is the standard deviation of n = 4). Dashes denote no data. This table shows data from Figure 3.

Figure 3, Panel (a) Mobilised Cu concentrations (µM) pH 6

| time (h) | CuS  no mb | range | CuS  mb | range | CuFeS_2_  no mb | range | CuFeS_2_  mb | range |
| --- | --- | --- | --- | --- | --- | --- | --- | --- |
| 0.167 | 2.4 | 0.7 | 1.9 | 0.3 | 0.5 | 0.2 | 1.3 | 0.6 |
| 0.5 | 2.7 | 0.3 | 1.2 | 0.8 | 0.9 | 0.1 | 2.5 | 0.0 |
| 1.0 | 2.7 | 0.3 | 3.4 | 0.8 | 0.7 | 0.2 | 1.7 | 0.1 |
| 2.0 | 2.7 | 0.2 | 3.7 | 0.5 | 0.5 | 0.1 | 2.3 | 0.0 |
| 4.0 | 2.8 | 0.5 | 4.1 | 0.2 | 0.5 | 0.1 | 2.5 | 0.2 |
| 8.0 | 3.4 | 0.4 | 5.5 | 0.4 | 0.5 | 0.0 | 4.5 | 0.8 |

Figure 3, Panel (b) Mobilised Cu concentrations (µM) pH 6.5

| time (h) | CuS  no mb | range | CuS  mb | range | CuFeS_2_  no mb | range | CuFeS_2_  mb | range |
| --- | --- | --- | --- | --- | --- | --- | --- | --- |
| 0.083 | - | - | 2.0 | 0.2 | - | - | - | - |
| 0.167 | 2.2 | 0.1 | - | - | 0.5 | 0.1 | 1.0 | 0.0 |
| 0.25 | - | - | 2.4 | 0.4 | - | - | - | - |
| 0.5 | 2.0 | 0.2 | 2.6 | 0.2 | 0.3 | 0.1 | 1.2 | 0.3 |
| 1.0 | 2.5 | 0.3 | 2.7 | 0.4 | 0.1 | 0.0 | 1.1 | 0.3 |
| 2.0 | 2.6 | 0.1 | 3.3 | 0.3 | 0.2 | 0.1 | 2.4 | 0.7 |
| 4.0 | 2.8 | 0.3 | 3.5 | 0.8 | 0.1 | 0.1 | 2.6 | 0.6 |
| 8.0 | 2.5 | 0.3 | 4.7 | 2.2 | 0.3 | 0.2 | 3.4 | 0.3 |

Figure 3, Panel (c) Mobilised Cu concentrations (µM) pH 7

| time (h) | CuS  no mb | range | CuS  mb | range | CuFeS_2_  no mb | range | CuFeS_2_  mb | range |
| --- | --- | --- | --- | --- | --- | --- | --- | --- |
| 0.083 | - | - | 0.3 | 0.1 | - | - | - | - |
| 0.167 | 1.5 | 0.0 | 1.8 | 0.2 | 0.0 | 0.0 | 0.6 | 0.2 |
| 0.25 | - | - | 0.5 | 0.3 | - | - | - | - |
| 0.5 | 2.1 | 0.2 | 1.4 | 1.2 | 0.0 | 0.0 | 0.6 | 0.2 |
| 1.0 | 1.3 | 0.0 | 2.9 | 3.7 | 0.0 | 0.0 | 0.7 | 0.3 |
| 2.0 | 2.1 | 0.2 | 2.0 | 1.8 | 0.0 | 0.0 | 1.2 | 0.1 |
| 4.0 | 2.2 | 0.2 | 2.7 | 1.4 | 0.0 | 0.0 | 1.3 | 0.2 |
| 8.0 | 2.9 | 0.4 | 2.6 | 2.0 | 0.0 | 0.0 | 1.7 | 0.0 |

Figure 3, Panel (d) Mobilised Cu concentrations (µM) pH 7.5

| time (h) | CuS  no mb | range | CuS  mb | range | CuFeS_2_  no mb | range | CuFeS_2_  mb | range |
| --- | --- | --- | --- | --- | --- | --- | --- | --- |
| 0.167 | 0.8 | 0.3 | 1.1 | 0.1 | 0.0 | 0.0 | 0.5 | 0.2 |
| 0.5 | 1.6 | 0.2 | 1.5 | 0.2 | 0.0 | 0.0 | 0.6 | 0.1 |
| 1.0 | 1.3 | 0.2 | 1.9 | 0.4 | 0.0 | 0.0 | 0.8 | 0.2 |
| 2.0 | 1.2 | 0.2 | 2.3 | 0.5 | 0.0 | 0.0 | 1.3 | 0.6 |
| 4.0 | 0.8 | 0.1 | 3.6 | 0.0 | 0.0 | 0.0 | 1.0 | 0.1 |
| 8.0 | 0.3 | 0.1 | 4.3 | 0.4 | 0.0 | 0.0 | 1.6 | 0.3 |

Figure 3, Panel (e) Mobilised Cu concentrations (µM) pH 8

| time (h) | CuS  no mb | range | CuS  mb | range | CuFeS_2_  no mb | range | CuFeS_2_  mb | range |
| --- | --- | --- | --- | --- | --- | --- | --- | --- |
| 0.167 | 0.8 | 0.2 | 1.9 | 0.9 | 0.0 | 0.0 | 0.7 | 0.0 |
| 0.5 | 1.3 | 0.1 | 2.8 | 0.2 | 0.3 | 0.1 | 1.9 | 0.0 |
| 1.0 | 1.6 | 0.0 | 2.5 | 0.5 | 0.1 | 0.0 | 1.2 | 0.2 |
| 2.0 | 1.0 | 0.0 | 3.5 | 0.2 | 0.2 | 0.1 | 1.4 | 0.3 |
| 4.0 | 0.8 | 0.4 | 4.2 | 0.2 | - | - | 1.5 | 0.2 |
| 8.0 | 0.2 | 0.1 | 4.5 | 0.9 | 0.2 | 0.1 | 2.1 | 0.2 |

Figure 3, Panel (f) Mobilised Cu concentrations (µM) pH 8.5

| time (h) | CuS  no mb | range | CuS  mb | range | CuFeS_2_  no mb | range | CuFeS_2_  mb | range |
| --- | --- | --- | --- | --- | --- | --- | --- | --- |
| 0.167 | 0.8 | 0.0 | 1.8 | 0.2 | 1.1 | 0.1 | 1.5 | 0.2 |
| 0.5 | 1.1 | 0.0 | 2.4 | 0.3 | 1.0 | 0.0 | 1.7 | 0.0 |
| 1.0 | 1.2 | 0.1 | 2.4 | 0.0 | 0.9 | 0.1 | 2.5 | 0.3 |
| 2.0 | 0.9 | 0.0 | 3.6 | 0.3 | 1.0 | 0.0 | 2.1 | 0.2 |
| 4.0 | 0.7 | 0.1 | 3.4 | 1.3 | - | - | - | - |
| 8.0 | 0.6 | 0.0 | 4.3 | 0.2 | 1.0 | 0.1 | 2.5 | 0.0 |

Data Table 4. Final (8 h) Cu and Fe concentrations (µM) in presence of 20 µM mb minus the control without mb in CuS and CuFeS_2_ dissolution experiments under (a) anoxic conditions and (b) oxic conditions from pH 6 – pH 8.5. The data is taken from that presented in Figures 3, 6, S4 and S5 for Cu and Figures 5, 7 and S6 for Fe. In this case the range is the pooled range of duplicate measurements of mb treatments and the control. Dashes denote no data. This table shows data from Figure 4.

Figure 4, Panel (a) Cu concentrations (µM), anoxic conditions

| pH | Cu (CuS) | range | Cu (CuFeS_2_) | range | Fe (CuFeS_2_) | range |
| --- | --- | --- | --- | --- | --- | --- |
| 6 | 2.1 | 0.4 | 4.2 | 0.6 | 2.5 | 2.0 |
| 6.5 | 2.0 | 1.6 | 3.1 | 0.2 | 1.0 | 2.7 |
| 7 | -0.3 | 1.4 | 1.6 | 0.0 | -2.0 | 1.0 |
| 7.5 | 4.0 | 0.3 | 1.5 | 0.2 | 2.4 | 0.7 |
| 8 | 4.2 | 0.7 | 1.9 | 0.2 | 0.1 | 1.4 |
| 8.5 | 3.8 | 0.2 | 1.5 | 0.0 | 1.4 | 0.8 |

Figure 4, Panel (b) Cu concentrations (µM), oxic conditions

| pH | Cu (CuS) | range | Cu (CuFeS_2_) | range | Fe (CuFeS_2_) | range |
| --- | --- | --- | --- | --- | --- | --- |
| 6 | 0.9 | 0.8 | 1.0 | 0.5 | 14.2 | 1.9 |
| 6.5 | -0.5 | 1.7 | 1.2 | 0.5 | 13.8 | 0.0 |
| 7 | 0.0 | 1.9 | 5.2 | 1.1 | 6.7 | 1.0 |
| 7.5 | 2.0 | 0.4 | 4.0 | 0.2 | 10.9 | 1.8 |
| 8 | 2.6 | 0.4 | 3.2 | 0.6 | 6.8 | 1.3 |
| 8.5 | 3.7 | 0.2 | 3.4 | 0.6 | 4.8 | 0.1 |

Data Table 5. Fe mobilised from chalcopyrite (2 g L^-1^, 10 mM NaCl) under anoxic conditions at (a) pH 6 (10 mM MES buffer), (b) 6.5 (10 mM MES buffer), (c) pH 7 (10 mM MOPS bueffer), (d) pH 7.5 (10 mM MOPS buffer), (e) pH 8 (10 mM PIPPS buffer) and (f) pH 8.5 (10 mM PIPPS buffer) in the absence and presence of 20 µM mb. The range is the range of duplicate measurements. Dashes denote no data. This table shows data from Figure 5.

Figure 5, Panel (a) Mobilised Fe concentrations (µM) pH 6

| time (h) | CuFeS_2_  no mb | range | CuFeS_2_  mb | range |
| --- | --- | --- | --- | --- |
| 0.167 | 5.9 | 1.7 | 3.5 | 1.0 |
| 0.5 | 5.2 | 0.1 | 4.9 | 0.1 |
| 1.0 | 5.8 | 1.8 | 4.5 | 0.0 |
| 2.0 | 6.8 | 2.0 | 7.2 | 0.7 |
| 4.0 | 10.0 | 1.7 | 7.1 | 0.2 |
| 8.0 | 13.3 | 1.8 | 15.8 | 1.2 |

Figure 5, Panel (b) Mobilised Fe concentrations (µM) pH 6.5

| time (h) | CuFeS_2_  no mb | range | CuFeS_2_  mb | range |
| --- | --- | --- | --- | --- |
| 0.167 | 6.3 | 1.5 | 4.9 | 0.2 |
| 0.5 | 6.0 | 2.2 | 5.3 | 0.5 |
| 1.0 | 6.4 | 0.3 | 5.1 | 1.1 |
| 2.0 | 8.1 | 0.3 | 8.4 | 0.5 |
| 4.0 | 9.6 | 1.1 | 11.2 | 3.5 |
| 8.0 | 12.3 | 2.9 | 13.3 | 2.5 |

Figure 5, Panel (c) Mobilised Fe concentrations (µM) pH 7

| time (h) | CuFeS_2_  no mb | range | CuFeS_2_  mb | range |
| --- | --- | --- | --- | --- |
| 0.167 | 2.0 | 0.3 | 2.5 | 0.2 |
| 0.5 | 2.6 | 0.3 | 4.5 | 2.4 |
| 1.0 | 2.7 | 0.2 | 3.5 | 0.1 |
| 2.0 | 3.5 | 0.1 | 5.0 | 0.0 |
| 4.0 | 4.1 | 0.2 | 4.7 | 0.1 |
| 8.0 | 7.8 | 1.0 | 5.8 | 0.1 |

Figure 5, Panel (d) Mobilised Fe concentrations (µM) pH 7.5

| time (h) | CuFeS_2_  no mb | range | CuFeS_2_  mb | range |
| --- | --- | --- | --- | --- |
| 0.167 | 1.5 | 0.3 | 3.0 | 0.4 |
| 0.5 | 1.5 | 0.1 | 3.3 | 1.4 |
| 1.0 | 1.8 | 0.4 | 3.5 | 1.3 |
| 2.0 | 2.5 | 0.1 | 4.0 | 0.9 |
| 4.0 | 2.4 | 0.4 | 4.2 | 0.8 |
| 8.0 | 2.8 | 0.2 | 5.3 | 0.9 |

Figure 5, Panel (e) Mobilised Fe concentrations (µM) pH 8

| time (h) | CuFeS_2_  no mb | range | CuFeS_2_  mb | range |
| --- | --- | --- | --- | --- |
| 0.167 | 0.6 | 0.0 | 3.0 | 1.1 |
| 0.5 | 1.4 | 0.2 | 3.9 | 0.1 |
| 1.0 | 1.4 | 0.0 | 2.8 | 0.5 |
| 2.0 | 2.0 | 0.2 | 5.5 | 0.1 |
| 4.0 | 2.7 | 2.2 | 4.2 | 0.0 |
| 8.0 | 4.3 | 1.9 | 4.4 | 0.0 |

Figure 5, Panel (f) Mobilised Fe concentrations (µM) pH 8.5

| time (h) | CuFeS_2_  no mb | range | CuFeS_2_  mb | range |
| --- | --- | --- | --- | --- |
| 0.167 | 5.4 | 1.3 | 4.9 | 0.4 |
| 0.5 | 4.3 | 0.3 | 4.8 | 0.2 |
| 1.0 | 6.3 | 2.9 | 5.7 | 0.2 |
| 2.0 | 4.6 | 0.3 | 6.0 | 0.1 |
| 8.0 | 5.4 | 0.5 | 6.8 | 1.0 |

Data Table 6. Cu mobilised from covellite at pH 6 (a) and pH 7.5 (c) and chalcopyrite at pH 6 (b) and pH 7.5 (d) (both 2 g L^-1^, 10 mM NaCl) under oxic conditions and under anoxic conditions in the absence and presence of 20 µM mb. The range is the range of duplicate measurements. Dashes denote no data. This table shows data from Figure 6.

Figure 6, Panel (a) Cu (µM) mobilised from covellite, pH 6

| time (h) | anoxic  no mb | range | anoxic  mb | range | 21% oxygen  no mb | range | 21% oxygen  mb | range |
| --- | --- | --- | --- | --- | --- | --- | --- | --- |
| 0.167 | 2.4 | 0.7 | 1.9 | 0.3 | 2.9 | 0.5 | 2.7 | 0.4 |
| 0.5 | 2.7 | 0.3 | 1.2 | 0.8 | 2.9 | 0.6 | 2.7 | 0.1 |
| 1.0 | 2.7 | 0.3 | 3.4 | 0.8 | 3.3 | 0.9 | 3.4 | 0.2 |
| 2.0 | 2.7 | 0.3 | 3.7 | 0.5 | 3.8 | 1.3 | 3.4 | 0.5 |
| 4.0 | 2.8 | 0.5 | 4.1 | 0.2 | 3.8 | 1.1 | 3.7 | 0.3 |
| 8.0 | 3.4 | 0.4 | 5.5 | 0.4 | 4.4 | 1.0 | 5.3 | 0.2 |

Figure 6, Panel (b) Cu (µM) mobilised from chalcopyrite, pH 6

| time (h) | anoxic  no mb | range | anoxic  mb | range | 21% oxygen  no mb | range | 21% oxygen  mb | range |
| --- | --- | --- | --- | --- | --- | --- | --- | --- |
| 0.083 | - | - | - | - | 3.7 | 0.2 | 3.6 | 0.2 |
| 0.167 | 0.5 | 0.2 | 1.3 | 0.6 | - | - | - | - |
| 0.25 | - | - | - | - | 4.5 | 0.5 | 3.9 | 0.3 |
| 0.5 | 0.9 | 0.1 | 2.5 | 0.0 | 4.8 | 0.0 | 4.5 | 0.2 |
| 1.0 | 0.7 | 0.2 | 1.7 | 0.1 | 5.9 | 0.8 | 6.0 | 0.2 |
| 2.0 | 0.5 | 0.1 | 2.3 | 0.0 | 5.8 | 0.1 | 6.5 | 0.4 |
| 4.0 | 0.5 | 0.1 | 2.5 | 0.2 | 7.3 | 0.2 | 7.6 | 0.6 |
| 8.0 | 0.5 | 0.0 | 4.5 | 0.8 | 7.6 | 0.6 | 8.5 | 0.4 |

Figure 6, Panel (c) Cu (µM) mobilised from covellite, pH 7.5

| time (h) | anoxic  no mb | range | anoxic  mb | range | 21% oxygen  no mb | range | 21% oxygen  mb | range |
| --- | --- | --- | --- | --- | --- | --- | --- | --- |
| 0.083 | - | - | - | - | - | - | 1.0 | 0.1 |
| 0.167 | 0.8 | 0.3 | 1.1 | 0.1 | 0.8 | 0.5 | - | - |
| 0.25 | - | - | - | - | - | - | 1.4 | 0.0 |
| 0.5 | 1.6 | 0.2 | 1.5 | 0.2 | 1.2 | 0.1 | 1.3 | 0.4 |
| 1.0 | 1.3 | 0.2 | 1.9 | 0.4 | 1.4 | 0.2 | 1.7 | 0.3 |
| 2.0 | 1.2 | 0.2 | 2.3 | 0.5 | 1.3 | 0.4 | 2.1 | 0.2 |
| 4.0 | 0.8 | 0.1 | 3.6 | 0.0 | 1.3 | 0.4 | 1.7 | 0.3 |
| 8.0 | 0.3 | 0.1 | 4.3 | 0.4 | 1.1 | 0.1 | 3.1 | 0.5 |

Figure 6, Panel (d) Cu (µM) mobilised from chalcopyrite, pH 7.5

| time (h) | anoxic  no mb | range | anoxic  mb | range | 21% oxygen  no mb | range | 21% oxygen  mb | range |
| --- | --- | --- | --- | --- | --- | --- | --- | --- |
| 0.083 | - | - | - | - | 0.8 | 0.0 | 1.9 | 0.0 |
| 0.167 | 0.0 | 0.0 | 0.5 | 0.2 | - | - | - | - |
| 0.25 | - | - | - | - | 0.8 | 0.0 | 2.4 | 0.5 |
| 0.5 | 0.0 | 0.0 | 0.6 | 0.1 | 0.7 | 0.0 | 2.2 | 0.4 |
| 1.0 | 0.0 | 0.0 | 0.8 | 0.2 | 0.8 | 0.0 | 2.5 | 0.1 |
| 2.0 | 0.0 | 0.0 | 1.3 | 0.6 | 0.9 | 0.0 | 3.0 | 0.1 |
| 4.0 | 0.0 | 0.0 | 1.0 | 0.1 | 0.9 | 0.1 | 4.1 | 0.4 |
| 8.0 | 0.0 | 0.0 | 1.6 | 0.3 | 0.9 | 0.1 | 5.0 | 0.3 |

Data Table 7. Fe mobilised from chalcopyrite at pH 6 (a) and pH 7.5 (b) (both 2 g L^-1^, 10 mM NaCl) under oxic conditions and under anoxic conditions in the absence and presence of 20 µM mb. The range is the range of duplicate measurements. Dashes denote no data. This table shows data from Figure 7.

Figure 7, Panel (a) Fe (µM) mobilised from chalcopyrite, pH 6

| time (h) | anoxic  no mb | range | anoxic  mb | range | 21% oxygen  no mb | range | 21% oxygen  mb | range |
| --- | --- | --- | --- | --- | --- | --- | --- | --- |
| 0.083 | - | - | - | - | 4.8 | 0.2 | 22.6 | 3.8 |
| 0.167 | 5.9 | 1.7 | 3.5 | 1.0 | - | - | - | - |
| 0.25 | - | - | - | - | 5.9 | 0.9 | 18.6 | 0.2 |
| 0.5 | 5.2 | 0.1 | 4.9 | 0.1 | 5.1 | 2.6 | 17.2 | 0.4 |
| 1.0 | 5.8 | 1.8 | 4.5 | 0.0 | 2.8 | 0.1 | - | - |
| 2.0 | 6.8 | 2.0 | 7.2 | 0.7 | 2.5 | 0.7 | 16.3 | 1.1 |
| 4.0 | 10.0 | 1.7 | 7.1 | 0.2 | 2.3 | 1.2 | 16.5 | 3.3 |
| 8.0 | 13.3 | 1.8 | 15.8 | 1.2 | 0.9 | 0.1 | 15.1 | 2.6 |

Figure 7, Panel (b) Fe (µM) mobilised from chalcopyrite, pH 7.5

| time (h) | anoxic  no mb | range | anoxic  mb | range | 21% oxygen  no mb | range | 21% oxygen  mb | range |
| --- | --- | --- | --- | --- | --- | --- | --- | --- |
| 0.083 | - | - | - | - | 1.1 | 0.3 | 14.9 | 1.2 |
| 0.167 | 1.5 | 0.3 | 3.0 | 0.4 | - | - | - | - |
| 0.25 | - | - | - | - | 1.3 | 0.8 | 12.4 | 0.9 |
| 0.5 | 1.5 | 0.1 | 3.3 | 1.4 | 1.6 | 1.2 | - | - |
| 1.0 | 1.8 | 0.4 | 3.5 | 1.3 | 2.1 | 1.5 | 15.5 | 3.6 |
| 2.0 | 2.5 | 0.1 | 4.0 | 0.9 | 2.6 | 1.7 | 16.7 | 8.1 |
| 4.0 | 2.4 | 0.4 | 4.2 | 0.8 | 0.8 | 0.3 | - | - |
| 8.0 | 2.8 | 0.2 | 5.3 | 0.9 | 0.9 | 0.3 | 11.8 | 2.6 |

Data Table 8. Cu mobilised from Cu_x_S NPs (10 mM NaCl) in the presence of Suwannee River fulvic acids (SRFA) and absence of SRFA at pH 7.5 under (a) low oxygen conditions (pO_2_ = 0.01 atm) and under high oxygen conditions (pO_2_ = 0.21 atm) in the presence and absence of 19 µM mb. The range is the range of duplicate measurements. This table shows data from Figure S2.

Figure S2, Panel (a) Mobilised Cu concentrations (µM) at 1% oxygen.

| time (h) | no SRFA  no mb | range | no SRFA mb | range | SRFA  no mb | range | SRFA  mb | range |
| --- | --- | --- | --- | --- | --- | --- | --- | --- |
| 1 | 0.4 | 0.2 | 5.7 | 0.0 | 6.2 | 0.1 | 7.3 | 0.1 |
| 4 | 0.1 | 0.0 | 7.1 | 0.4 | 5.5 | 0.0 | 6.7 | 0.2 |
| 8 | 0.1 | 0.0 | 8.4 | 1.3 | 6.8 | 0.1 | 6.9 | 0.3 |
| 24 | 0.1 | 0.0 | 6.2 | 0.1 | 7.1 | 0.2 | 6.2 | 1.5 |
| 72 | 0.3 | 0.1 | 8.6 | 0.7 | 6.2 | 0.2 | 8.3 | 2.9 |
| 168 | 0.7 | 0.4 | 8.4 | 1.6 | 6.3 | 0.0 | 8.9 | 0.5 |
| 528 | 0.2 | 0.0 | 15.3 | 0.6 | 12.2 | 0.8 | 11.1 | 0.8 |

Figure S2, Panel (b) Mobilised Cu concentrations (µM) at 21% oxygen.

| time (h) | SRFA  no mb | range | SRFA  mb | range |
| --- | --- | --- | --- | --- |
| 1 | 1.0 | 0.0 | 10.9 | 0.3 |
| 4 | 2.2 | 0.3 | 10.9 | 1.9 |
| 8 | 3.1 | 0.1 | 14.8 | 1.0 |
| 24 | 9.8 | 1.4 | 16.6 | 0.6 |
| 72 | 10.5 | 1.5 | 21.1 | 1.0 |
| 168 | 10.5 | 0.2 | 22.4 | 1.7 |
| 528 | 9.2 | 0.2 | 20.9 | 0.9 |

Data Table 9. Cu mobilised from Cu_x_S NPs (10 mM NaCl) in the presence of Suwannee River fulvic acids (SRFA) and absence of SRFA at pH 7.5 at pH 5, pH 6.5, pH 7.5 and pH 8.5 under (a) anoxic conditions and (b) low oxygen conditions (pO2 = 0.01 atm) in the presence and absence of 19 µM mb. The range is the range of duplicate measurements. This table shows data from Figure S3.

Figure S3, Panel (a) Mobilised Cu concentrations (µM) anoxic.

| time (h) | pH 5 no mb | range | pH 5 mb | range | pH 6 no mb | range | pH 6 mb | range | pH 7.5 no mb | range | pH 7.5 mb | range | pH 8.5 no mb | range | pH 8.5 mb | range |
| --- | --- | --- | --- | --- | --- | --- | --- | --- | --- | --- | --- | --- | --- | --- | --- | --- |
| 1 | 0.3 | 0.0 | 0.5 | 0.0 | 0.0 | 0.0 | 5.4 | 1.4 | 0.1 | 0.0 | 7.3 | 0.2 | 0.3 | 0.0 | 4.7 | 0.0 |
| 4 | 0.3 | 0.0 | 0.5 | 0.2 | 0.4 | 0.0 | 6.2 | 1.1 | 0.1 | 0.0 | 6.8 | 0.2 | 0.4 | 0.0 | 5.9 | 0.4 |
| 8 | 0.5 | 0.1 | 0.6 | 0.0 | 0.5 | 0.0 | 5.5 | 0.0 | 0.1 | 0.9 | 6.9 | 0.7 | 1.2 | 0.0 | 6.7 | 0.0 |
| 24 | 0.8 | 0.1 | 0.9 | 0.0 | 0.7 | 0.3 | 7.6 | 0.7 | 0.2 | 0.1 | 6.2 | 0.4 | 0.4 | 0.0 | 6.8 | 0.0 |
| 72 | 1.6 | 0.2 | 1.2 | 0.2 | 5.6 | 0.6 | 7.3 | 0.7 | 0.3 | 0.1 | 8.3 | 0.6 | 0.7 | 0.0 | 8.6 | 0.4 |
| 168 | 1.6 | 0.1 | 0.8 | 0.4 | 3.3 | 0.9 | 7.3 | 0.2 | 0.3 | 0.0 | 8.9 | 1.3 | 0.9 | 0.1 | 7.2 | 1.5 |

Figure S3, Panel (b) Mobilised Cu concentrations (µM) at 1% oxygen.

| time (h) | pH 5 no mb | range | pH 5 mb | range | pH 6 no mb | range | pH 6 mb | range | pH 7.5 no mb | range | pH 7.5 mb | range | pH 8.5 no mb | range | pH 8.5 mb | range |
| --- | --- | --- | --- | --- | --- | --- | --- | --- | --- | --- | --- | --- | --- | --- | --- | --- |
| 1 | 0.8 | 0.7 | 1.5 | 1.4 | 0.7 | 0.1 | 4.6 | 0.4 | 6.2 | 0.1 | 7.3 | 0.3 | 0.4 | 0.1 | 5.4 | 0.1 |
| 4 | 0.3 | 0.0 | 1.2 | 0.9 | 0.4 | 0.0 | 4.5 | 0.1 | 5.5 | 0.0 | 6.8 | 0.4 | 0.4 | 0.0 | 6.7 | 0.2 |
| 8 | 0.6 | 0.1 | 1.4 | 0.9 | 0.6 | 0.0 | 6.7 | 0.1 | 6.8 | 0.0 | 6.9 | 0.4 | 0.6 | 0.1 | 5.5 | 0.3 |
| 24 | 1.9 | 0.4 | 2.2 | 1.1 | 1.9 | 0.5 | 6.2 | 0.5 | 7.1 | 0.0 | 6.2 | 0.7 | 0.8 | 0.2 | 7.5 | 1.5 |
| 72 | 4.2 | 0.0 | 3.0 | 0.2 | 2.4 | 1.0 | 7.8 | 1.0 | 6.2 | 0.1 | 8.3 | 0.4 | 1.5 | 0.2 | 10.7 | 2.9 |
| 168 | 8.5 | 0.6 | 6.8 | 0.0 | 4.0 | 0.1 | 11.3 | 0.8 | 6.3 | 0.5 | 8.9 | 0.7 | 2.8 | 0.0 | 10.0 | 0.5 |

Data Table 10. Cu (µM) mobilised from covellite (2 g L^-1^, 10 mM NaCl) under oxic conditions (pO_2_ = 0.21 atm) and under anoxic conditions at (a) pH 6.5 (b) pH 7 (c) pH 8 and (d) pH 8.5 in absence and presence of 20 µM mb. The range is the range of duplicate measurements. Dashes denote no data. This table shows data from Figure S4.

Figure S4, Panel (a) Cu concentrations (µM), pH 6.5.

| time (h) | anoxic  no mb | range | anoxic  mb | range | 21% oxygen  no mb | range | 21% oxygen mb | range |
| --- | --- | --- | --- | --- | --- | --- | --- | --- |
| 0.083 | - | - | - | - | - | - | 2.0 | 0.2 |
| 0.167 | 2.2 | 0.1 | 2.6 | 1.2 | 3.2 | 0.3 | - | - |
| 0.25 | - | - | - | - | - | - | 2.4 | 0.4 |
| 0.5 | 2.0 | 0.2 | 2.5 | 0.6 | 3.6 | 0.2 | 2.6 | 0.2 |
| 1.0 | 2.5 | 0.3 | 2.2 | 0.2 | 4.3 | 0.5 | 2.7 | 0.4 |
| 2.0 | 2.6 | 0.1 | 2.7 | 0.7 | 4.4 | 0.4 | 3.3 | 0.3 |
| 4.0 | 2.8 | 0.3 | 3.6 | 0.5 | 4.5 | 0.2 | 3.5 | 0.8 |
| 8.0 | 2.5 | 0.3 | 4.5 | 1.3 | 5.2 | 0.9 | 4.7 | 2.2 |

Figure S4, Panel (b) Cu concentrations (µM), pH 7.

| time (h) | anoxic  no mb | range | anoxic  mb | range | 21% oxygen no mb | range | 21% oxygen mb | range |
| --- | --- | --- | --- | --- | --- | --- | --- | --- |
| 0.083 | - | - | 0.3 | 0.1 | - | - | - | - |
| 0.167 | 1.5 | 0.0 | 1.8 | 0.2 | 1.1 | 0.5 | 0.5 | 0.5 |
| 0.25 | - | - | 0.5 | 0.3 | - | - | - | - |
| 0.5 | 2.1 | 0.2 | 1.4 | 1.2 | 2.2 | 0.5 | 0.5 | 0.2 |
| 1.0 | 2.4 | 1.6 | 2.9 | 3.7 | 1.6 | 0.7 | 1.0 | 0.1 |
| 2.0 | 2.1 | 0.2 | 2.0 | 1.8 | 1.8 | 0.8 | 1.5 | 0.1 |
| 4.0 | 2.2 | 0.2 | 2.7 | 1.4 | 2.7 | 0.5 | 2.8 | 0.4 |
| 8.0 | 2.9 | 0.4 | 2.6 | 2.0 | 3.7 | 2.6 | 3.8 | 0.4 |

Figure S4, Panel (c) Cu concentrations (µM), pH 8.

| time (h) | anoxic  no mb | range | anoxic  mb | range | 21% oxygen no mb | range | 21% oxygen mb | range |
| --- | --- | --- | --- | --- | --- | --- | --- | --- |
| 0.083 | - | - | - | - | 1.6 | 0.1 | 1.1 | 0.0 |
| 0.167 | 0.8 | 0.2 | 1.9 | 0.9 | - | - | - | - |
| 0.25 | - | - | - | - | 1.5 | 0.2 | 1.5 | 0.1 |
| 0.5 | 1.3 | 0.1 | 2.8 | 0.2 | 1.4 | 0.0 | 1.3 | 0.2 |
| 1.0 | 1.6 | 0.0 | 2.5 | 0.5 | 1.5 | 0.1 | 1.8 | 0.1 |
| 2.0 | 1.0 | 0.0 | 3.5 | 0.2 | 0.7 | 0.2 | 2.3 | 0.5 |
| 4.0 | 0.8 | 0.4 | 4.2 | 0.2 | 0.5 | 0.2 | 2.2 | 0.4 |
| 8.0 | 0.2 | 0.1 | 4.5 | 0.9 | 0.5 | 0.0 | 3.0 | 0.5 |

Figure S4, Panel (d) Cu concentrations (µM), pH 8.5.

| time (h) | anoxic  no mb | range | anoxic  mb | range | 21% oxygen no mb | range | 21% oxygen mb | range |
| --- | --- | --- | --- | --- | --- | --- | --- | --- |
| 0.167 | 0.8 | 0.0 | 1.8 | 0.2 | 0.1 | 0.2 | 0.3 | 0.2 |
| 0.5 | 1.1 | 0.0 | 2.4 | 0.3 | 0.1 | 0.1 | 0.5 | 0.1 |
| 1.0 | 1.2 | 0.1 | 2.4 | 0.0 | 0.1 | 0.1 | 1.1 | 0.2 |
| 2.0 | 0.9 | 0.0 | 3.6 | 0.3 | 0.1 | 0.1 | 1.3 | 0.0 |
| 4.0 | 0.7 | 0.1 | 3.4 | 1.3 | 0.1 | 0.2 | 2.6 | 0.4 |
| 8.0 | 0.6 | 0.0 | 4.3 | 0.2 | 0.0 | 0.0 | 3.7 | 0.2 |

Data Table 11. Cu (µM) mobilised from chalcopyrite (2 g L^-1^, 10 mM NaCl) under oxic conditions (pO_2_ = 0.21 atm) and under anoxic conditions at (a) pH 6.5 (b) pH 7 (c) pH 8 and (d) pH 8.5 in absence and presence of 20 µM mb. The range is the range of duplicate measurements. Dashes denote no data. This table shows data from Figure S5.

Figure S5, Panel (a) Cu concentrations (µM), pH 6.5.

| time (h) | anoxic  no mb | range | anoxic  mb | range | 21% oxygen  no mb | range | 21% oxygen mb | range |
| --- | --- | --- | --- | --- | --- | --- | --- | --- |
| 0.083 | - | - | - | - | 1.3 | 0.0 | - | - |
| 0.167 | 0.5 | 0.1 | 1.0 | 0.0 | - | - | 2.3 | 0.1 |
| 0.25 | - | - | - | - | 1.1 | 0.3 | - | - |
| 0.5 | 0.3 | 0.1 | 1.2 | 0.3 | 1.4 | 0.0 | 2.8 | 0.1 |
| 1.0 | 0.1 | 0.0 | 1.1 | 0.3 | 1.9 | 0.0 | 3.2 | 0.1 |
| 2.0 | 0.2 | 0.1 | 2.4 | 0.7 | 2.5 | 0.2 | 3.7 | 0.0 |
| 4.0 | 0.1 | 0.1 | 2.6 | 0.6 | 3.6 | 0.1 | 4.3 | 0.2 |
| 8.0 | 0.3 | 0.2 | 3.4 | 0.3 | 4.0 | 0.1 | 5.2 | 0.7 |

Figure S5, Panel (b) Cu concentrations (µM), pH 7.

| time (h) | anoxic  no mb | range | anoxic  mb | range | 21% oxygen no mb | range | 21% oxygen mb | range |
| --- | --- | --- | --- | --- | --- | --- | --- | --- |
| 0.083 | - | - | - | - | 0.0 | 0.0 | 0.6 | 0.2 |
| 0.167 | 0.0 | 0.0 | 0.6 | 0.2 | - | - | - | - |
| 0.25 | - | - | - | - | 0.0 | 0.0 | 0.8 | 0.3 |
| 0.5 | 0.0 | 0.0 | 0.6 | 0.2 | 0.0 | 0.1 | 1.1 | 0.2 |
| 1.0 | 0.0 | 0.0 | 0.7 | 0.3 | 0.0 | 0.0 | 1.5 | 0.3 |
| 2.0 | 0.0 | 0.0 | 1.2 | 0.1 | 0.1 | 0.0 | 2.0 | 0.2 |
| 4.0 | 0.0 | 0.0 | 1.3 | 0.2 | 0.2 | 0.0 | 2.9 | 0.1 |
| 8.0 | 0.0 | 0.0 | 1.7 | 0.0 | 0.3 | 0.0 | 5.5 | 1.5 |

Figure S5, Panel (c) Cu concentrations (µM), pH 8.

| time (h) | anoxic  no mb | range | anoxic  mb | range | 21% oxygen no mb | range | 21% oxygen mb | range |
| --- | --- | --- | --- | --- | --- | --- | --- | --- |
| 0.083 | - | - | - | - | 1.4 | 0.0 | 2.4 | 0.0 |
| 0.167 | 0.0 | 0.0 | 1.5 | 1.1 | - | - | - | - |
| 0.25 | - | - | - | - | 1.4 | 0.1 | 2.2 | 0.0 |
| 0.5 | 0.3 | 0.1 | 2.7 | 1.2 | 1.4 | 0.0 | 2.5 | 0.1 |
| 1.0 | 0.0 | 0.0 | 1.2 | 0.2 | 1.5 | 0.0 | 3.0 | 0.4 |
| 2.0 | 0.2 | 0.1 | 1.4 | 0.3 | 1.4 | 0.1 | 3.1 | 0.5 |
| 4.0 | 1.8 | 2.6 | 1.5 | 0.2 | 1.5 | 0.0 | 3.9 | 0.5 |
| 8.0 | 0.2 | 0.1 | 2.1 | 0.2 | 1.6 | 0.0 | 4.8 | 0.9 |

Figure S5, Panel (d) Cu concentrations (µM), pH 8.5.

| time (h) | anoxic  no mb | range | anoxic  mb | range | 21% oxygen no mb | range | 21% oxygen mb | range |
| --- | --- | --- | --- | --- | --- | --- | --- | --- |
| 0.083 | - | - | - | - | 1.6 | 0.0 | 2.4 | 0.3 |
| 0.167 | 1.1 | 0.1 | 1.5 | 0.2 | - | - | - | - |
| 0.25 | - | - | - | - | 1.5 | 0.1 | 2.3 | 0.1 |
| 0.5 | 1.0 | 0.0 | 1.7 | 0.0 | 1.5 | 0.0 | 2.6 | 0.3 |
| 1.0 | 0.9 | 0.1 | 2.5 | 0.3 | 1.6 | 0.0 | 2.7 | 0.3 |
| 2.0 | 1.0 | 0.0 | 2.1 | 0.2 | 1.6 | 0.0 | 3.0 | 0.3 |
| 4.0 | - | - | - | - | 1.7 | 0.1 | 3.6 | 0.3 |
| 8.0 | 1.0 | 0.1 | 2.5 | 0.0 | 1.7 | 0.1 | 5.1 | 0.8 |

Data Table 12. Fe (µM) mobilised from chalcopyrite (2 g L^-1^, 10 mM NaCl) under oxic conditions (pO_2_ = 0.21 atm) and under anoxic conditions at (a) pH 6.5 (b) pH 7 (c) pH 8 and (d) pH 8.5 in absence and presence of 20 µM mb. The range is the range of duplicate measurements. Dashes denote no data. This table shows data from Figure S6.

Figure S6, Panel (a) Fe concentrations (µM), pH 6.5.

| time (h) | anoxic  no mb | range | anoxic  mb | range | 21% oxygen  no mb | range | 21% oxygen mb | range |
| --- | --- | --- | --- | --- | --- | --- | --- | --- |
| 0.083 | - | - | - | - | 4.0 | 0.1 | 26.9 | 10.4 |
| 0.167 | 6.3 | 1.5 | 4.9 | 0.2 | - | - | - | - |
| 0.25 | - | - | - | - | 3.7 | 1.4 | 35.3 | 24.3 |
| 0.5 | 6.0 | 2.2 | 5.3 | 0.5 | 2.6 | 0.2 | 20.4 | 1.0 |
| 1.0 | 6.4 | 0.3 | 5.1 | 1.1 | 2.1 | 0.0 | 18.0 | 2.6 |
| 2.0 | 8.1 | 0.3 | 8.4 | 0.5 | 1.3 | 0.0 | 23.7 | 6.9 |
| 4.0 | 9.6 | 1.1 | 11.2 | 3.5 | 1.0 | 0.1 | 8.9 | 2.5 |
| 8.0 | 12.3 | 2.9 | 13.3 | 2.5 | 0.8 | 0.0 | 14.6 | 8.4 |

Figure S6, Panel (b) Fe concentrations (µM), pH 7.

| time (h) | anoxic  no mb | range | anoxic  mb | range | 21% oxygen no mb | range | 21% oxygen mb | range |
| --- | --- | --- | --- | --- | --- | --- | --- | --- |
| 0.083 | - | - | - | - | 5.3 | 4.4 | 3.1 | 0.2 |
| 0.167 | 2.0 | 0.3 | 2.5 | 0.2 | - | - | - | - |
| 0.25 | - | - | - | - | 3.8 | 1.1 | 4.9 | 1.3 |
| 0.5 | 2.6 | 0.3 | 4.5 | 2.4 | 2.2 | 1.8 | 0.6 | 0.1 |
| 1.0 | 2.7 | 0.2 | 3.5 | 0.1 | 1.2 | 0.3 | 5.0 | 2.0 |
| 2.0 | 3.5 | 0.1 | 5.0 | 0.0 | 1.3 | 0.4 | 3.0 | 0.4 |
| 4.0 | 4.1 | 0.2 | 4.7 | 0.1 | 4.6 | 0.7 | 4.8 | 1.3 |
| 8.0 | 7.8 | 1.0 | 5.8 | 0.1 | 1.8 | 1.1 | 8.5 | 1.2 |

Figure S6, Panel (c) Cu concentrations (µM), pH 8.

| time (h) | anoxic  no mb | range | anoxic  mb | range | 21% oxygen no mb | range | 21% oxygen mb | range |
| --- | --- | --- | --- | --- | --- | --- | --- | --- |
| 0.083 | - | - | - | - | 0.8 | 0.2 | 9.1 | 2.2 |
| 0.167 | 2.1 | 2.1 | 3.0 | 1.1 | - | - | - | - |
| 0.25 | - | - | - | - | 0.7 | 0.1 | 9.6 | 2.1 |
| 0.5 | 1.4 | 0.2 | 3.9 | 0.1 | 0.8 | 0.2 | 9.4 | 1.3 |
| 1.0 | 1.4 | 0.0 | 2.8 | 0.5 | 0.9 | 0.1 | 8.9 | 0.6 |
| 2.0 | 2.0 | 0.2 | 5.5 | 0.1 | 1.1 | 0.3 | 7.7 | 0.8 |
| 4.0 | 2.7 | 2.2 | 4.2 | 0.0 | 0.8 | 0.3 | 6.0 | 0.5 |
| 8.0 | 4.3 | 1.9 | 6.5 | 3.0 | 0.6 | 0.1 | 7.4 | 2.0 |

Figure S6, Panel (d) Cu concentrations (µM), pH 8.5.

| time (h) | anoxic  no mb | range | anoxic  mb | range | 21% oxygen no mb | range | 21% oxygen mb | range |
| --- | --- | --- | --- | --- | --- | --- | --- | --- |
| 0.083 | - | - | - | - | 1.2 | 0.5 | 11.8 | 3.9 |
| 0.167 | 5.4 | 1.3 | 4.9 | 0.4 | - | - | - | - |
| 0.25 | - | - | - | - | 2.3 | 1.8 | 12.5 | 0.0 |
| 0.5 | 4.3 | 0.3 | 4.8 | 0.2 | 1.0 | 0.3 | 9.6 | 2.5 |
| 1.0 | 6.3 | 2.9 | 5.7 | 0.2 | 0.8 | 0.3 | 7.5 | 0.8 |
| 2.0 | 4.6 | 0.3 | 6.0 | 0.1 | 1.1 | 0.4 | 7.1 | 0.9 |
| 4.0 | - | - | - | - | 0.8 | 0.0 | 7.1 | 0.8 |
| 8.0 | 5.4 | 0.5 | 6.8 | 1.0 | 1.1 | 0.2 | 7.9 | 3.0 |

Data Table 13. Fe concentrations mobilised from chalcopyrite after 8 hours under anoxic conditions in the absence of mb (2 g L^-1^ solids, 10 mM NaCl) and Fe concentrations in equilibrium with chamosite calculated using PHREEQC (10 mM NaCl), all as a function of pH. The range is the range of duplicate measurements. Dashes denote no data. This table shows data from Figure S7.

Figure S7, Fe concentrations (µM) mobilised from chalcopyrite and in equilibrium with chamosite.

| pH | chalcopyrite no mb | range | chamosite model |
| --- | --- | --- | --- |
| 6 | 13.3 | 1.8 | 53.2 |
| 6.1 | - | - | 41.1 |
| 6.2 | - | - | 32.2 |
| 6.3 | - | - | 25.5 |
| 6.4 | - | - | 20.6 |
| 6.5 | 12.3 | 2.9 | 16.9 |
| 6.6 | - | - | 14.2 |
| 6.7 | - | - | 12.0 |
| 6.8 | - | - | 10.4 |
| 6.9 | - | - | 9.1 |
| 7 | 7.8 | 1.0 | 8.0 |
| 7.1 | - | - | 7.1 |
| 7.2 | - | - | 6.4 |
| 7.3 | - | - | 5.8 |
| 7.4 | - | - | 5.2 |
| 7.5 | 2.8 | 0.2 | 4.7 |
| 7.6 | - | - | 4.3 |
| 7.7 | - | - | 3.9 |
| 7.8 | - | - | 3.5 |
| 7.9 | - | - | 3.2 |
| 8 | 4.3 | 1.9 | 2.9 |
| 8.1 | - | - | 2.7 |
| 8.2 | - | - | 2.5 |
| 8.3 | - | - | 2.2 |
| 8.4 | - | - | 2.1 |
| 8.5 | 5.4 | 0.5 | 1.9 |

Data Table 14. (a) UV-visible absorbance of 20 µM mb in the presence of various Fe(II) concentrations up to a 1:1 molar ratio (pH 7.5, 10 mM NaCl) prepared under anoxic conditions. (b) UV-visible absorbance of 20 µM mb at 361 nm as a function of Fe(II) (pH 7.5, 10 mM NaCl). This table shows data from Figure S8.

Figure S8, Panel (a) absorbance of 20 µM mb with varying Fe(II) concentrations.

| wavelength | 0 µM  Fe(II) | 2 µM Fe(II) | 2.5 µM Fe(II) | 5 µM Fe(II) | 7.5 µM Fe(II) | 10 µM Fe(II) |
| --- | --- | --- | --- | --- | --- | --- |
| 460.0 | 0.01 | 0.01 | 0.01 | 0.02 | 0.01 | 0.02 |
| 459.1 | 0.01 | 0.01 | 0.01 | 0.02 | 0.02 | 0.02 |
| 458.0 | 0.01 | 0.02 | 0.02 | 0.02 | 0.02 | 0.02 |
| 456.9 | 0.01 | 0.02 | 0.02 | 0.02 | 0.02 | 0.02 |
| 456.0 | 0.01 | 0.02 | 0.02 | 0.02 | 0.02 | 0.02 |
| 454.9 | 0.02 | 0.02 | 0.02 | 0.02 | 0.02 | 0.02 |
| 454.0 | 0.02 | 0.02 | 0.02 | 0.02 | 0.02 | 0.02 |
| 453.0 | 0.02 | 0.02 | 0.02 | 0.02 | 0.02 | 0.02 |
| 452.0 | 0.02 | 0.02 | 0.02 | 0.02 | 0.02 | 0.02 |
| 451.0 | 0.02 | 0.02 | 0.02 | 0.02 | 0.02 | 0.03 |
| 450.1 | 0.02 | 0.02 | 0.02 | 0.02 | 0.02 | 0.03 |
| 449.0 | 0.02 | 0.02 | 0.02 | 0.03 | 0.03 | 0.03 |
| 448.1 | 0.02 | 0.02 | 0.02 | 0.03 | 0.03 | 0.03 |
| 447.0 | 0.02 | 0.03 | 0.03 | 0.03 | 0.03 | 0.03 |
| 446.1 | 0.02 | 0.03 | 0.03 | 0.03 | 0.03 | 0.03 |
| 445.0 | 0.03 | 0.03 | 0.03 | 0.03 | 0.03 | 0.03 |
| 443.9 | 0.03 | 0.03 | 0.03 | 0.03 | 0.03 | 0.04 |
| 443.0 | 0.03 | 0.03 | 0.03 | 0.04 | 0.04 | 0.04 |
| 441.9 | 0.03 | 0.03 | 0.03 | 0.04 | 0.04 | 0.04 |
| 441.0 | 0.03 | 0.04 | 0.04 | 0.04 | 0.04 | 0.04 |
| 439.9 | 0.04 | 0.04 | 0.04 | 0.04 | 0.04 | 0.04 |
| 439.0 | 0.04 | 0.04 | 0.04 | 0.05 | 0.05 | 0.05 |
| 437.9 | 0.04 | 0.05 | 0.05 | 0.05 | 0.05 | 0.05 |
| 437.0 | 0.05 | 0.05 | 0.05 | 0.05 | 0.05 | 0.05 |
| 436.0 | 0.05 | 0.05 | 0.05 | 0.06 | 0.06 | 0.06 |
| 435.0 | 0.06 | 0.06 | 0.06 | 0.06 | 0.06 | 0.06 |
| 434.0 | 0.06 | 0.06 | 0.06 | 0.07 | 0.07 | 0.07 |
| 433.0 | 0.07 | 0.07 | 0.07 | 0.07 | 0.07 | 0.07 |
| 432.0 | 0.07 | 0.07 | 0.07 | 0.07 | 0.07 | 0.07 |
| 431.0 | 0.08 | 0.08 | 0.08 | 0.08 | 0.08 | 0.08 |
| 430.0 | 0.08 | 0.09 | 0.09 | 0.09 | 0.08 | 0.08 |
| 429.0 | 0.09 | 0.09 | 0.09 | 0.09 | 0.09 | 0.09 |
| 428.0 | 0.10 | 0.10 | 0.10 | 0.10 | 0.10 | 0.10 |
| 427.0 | 0.11 | 0.11 | 0.11 | 0.10 | 0.10 | 0.10 |
| 426.0 | 0.12 | 0.12 | 0.11 | 0.11 | 0.11 | 0.10 |
| 425.0 | 0.13 | 0.12 | 0.12 | 0.12 | 0.11 | 0.11 |
| 424.0 | 0.14 | 0.14 | 0.13 | 0.13 | 0.12 | 0.12 |
| 423.0 | 0.15 | 0.15 | 0.14 | 0.14 | 0.13 | 0.13 |
| 422.0 | 0.16 | 0.15 | 0.15 | 0.15 | 0.14 | 0.13 |
| 421.0 | 0.16 | 0.16 | 0.16 | 0.15 | 0.14 | 0.14 |
| 420.0 | 0.18 | 0.17 | 0.17 | 0.16 | 0.15 | 0.15 |
| 419.0 | 0.19 | 0.18 | 0.18 | 0.17 | 0.16 | 0.15 |
| 418.0 | 0.20 | 0.20 | 0.19 | 0.18 | 0.17 | 0.16 |
| 417.0 | 0.21 | 0.21 | 0.20 | 0.19 | 0.18 | 0.17 |
| 416.0 | 0.22 | 0.22 | 0.21 | 0.20 | 0.18 | 0.18 |
| 415.0 | 0.24 | 0.23 | 0.22 | 0.21 | 0.19 | 0.18 |
| 414.0 | 0.25 | 0.24 | 0.24 | 0.22 | 0.20 | 0.19 |
| 413.0 | 0.27 | 0.25 | 0.25 | 0.23 | 0.21 | 0.20 |
| 412.0 | 0.28 | 0.27 | 0.26 | 0.24 | 0.22 | 0.21 |
| 411.0 | 0.29 | 0.27 | 0.27 | 0.25 | 0.23 | 0.22 |
| 410.0 | 0.30 | 0.28 | 0.28 | 0.26 | 0.24 | 0.22 |
| 409.0 | 0.31 | 0.30 | 0.29 | 0.27 | 0.25 | 0.23 |
| 408.0 | 0.32 | 0.31 | 0.30 | 0.28 | 0.26 | 0.24 |
| 407.0 | 0.33 | 0.32 | 0.31 | 0.29 | 0.26 | 0.25 |
| 406.0 | 0.34 | 0.32 | 0.32 | 0.29 | 0.27 | 0.25 |
| 405.0 | 0.35 | 0.33 | 0.32 | 0.30 | 0.28 | 0.26 |
| 403.9 | 0.35 | 0.34 | 0.33 | 0.31 | 0.28 | 0.27 |
| 403.0 | 0.36 | 0.35 | 0.34 | 0.31 | 0.29 | 0.27 |
| 401.9 | 0.37 | 0.35 | 0.35 | 0.32 | 0.30 | 0.28 |
| 401.0 | 0.37 | 0.36 | 0.35 | 0.33 | 0.30 | 0.29 |
| 399.9 | 0.38 | 0.36 | 0.36 | 0.33 | 0.31 | 0.29 |
| 399.0 | 0.38 | 0.37 | 0.36 | 0.34 | 0.31 | 0.30 |
| 397.9 | 0.39 | 0.37 | 0.37 | 0.34 | 0.32 | 0.30 |
| 397.0 | 0.39 | 0.38 | 0.37 | 0.35 | 0.32 | 0.31 |
| 396.1 | 0.39 | 0.38 | 0.37 | 0.35 | 0.33 | 0.31 |
| 395.0 | 0.39 | 0.38 | 0.37 | 0.35 | 0.33 | 0.32 |
| 394.1 | 0.39 | 0.38 | 0.37 | 0.35 | 0.33 | 0.32 |
| 393.0 | 0.39 | 0.38 | 0.37 | 0.36 | 0.34 | 0.33 |
| 392.1 | 0.39 | 0.38 | 0.37 | 0.36 | 0.34 | 0.33 |
| 391.0 | 0.38 | 0.38 | 0.37 | 0.36 | 0.34 | 0.33 |
| 390.0 | 0.38 | 0.38 | 0.37 | 0.36 | 0.35 | 0.33 |
| 389.0 | 0.38 | 0.38 | 0.37 | 0.36 | 0.35 | 0.34 |
| 388.0 | 0.38 | 0.37 | 0.37 | 0.36 | 0.35 | 0.34 |
| 386.9 | 0.37 | 0.37 | 0.37 | 0.36 | 0.35 | 0.34 |
| 386.0 | 0.37 | 0.37 | 0.36 | 0.36 | 0.35 | 0.34 |
| 384.9 | 0.36 | 0.36 | 0.36 | 0.36 | 0.35 | 0.34 |
| 384.0 | 0.35 | 0.36 | 0.35 | 0.35 | 0.35 | 0.34 |
| 382.9 | 0.35 | 0.35 | 0.35 | 0.35 | 0.35 | 0.34 |
| 382.0 | 0.34 | 0.35 | 0.35 | 0.35 | 0.35 | 0.34 |
| 381.1 | 0.33 | 0.34 | 0.34 | 0.34 | 0.34 | 0.34 |
| 380.0 | 0.33 | 0.34 | 0.34 | 0.34 | 0.34 | 0.34 |
| 379.1 | 0.33 | 0.33 | 0.33 | 0.34 | 0.34 | 0.34 |
| 378.0 | 0.32 | 0.33 | 0.33 | 0.33 | 0.34 | 0.34 |
| 377.0 | 0.31 | 0.32 | 0.32 | 0.33 | 0.34 | 0.34 |
| 376.0 | 0.30 | 0.31 | 0.31 | 0.32 | 0.33 | 0.33 |
| 375.0 | 0.29 | 0.31 | 0.31 | 0.32 | 0.33 | 0.33 |
| 373.9 | 0.29 | 0.30 | 0.30 | 0.32 | 0.33 | 0.33 |
| 373.0 | 0.28 | 0.30 | 0.30 | 0.31 | 0.32 | 0.32 |
| 371.9 | 0.27 | 0.29 | 0.29 | 0.31 | 0.32 | 0.32 |
| 371.0 | 0.27 | 0.29 | 0.29 | 0.31 | 0.32 | 0.32 |
| 370.1 | 0.27 | 0.28 | 0.29 | 0.30 | 0.32 | 0.32 |
| 369.0 | 0.26 | 0.28 | 0.28 | 0.30 | 0.31 | 0.32 |
| 368.0 | 0.26 | 0.27 | 0.28 | 0.30 | 0.31 | 0.32 |
| 367.0 | 0.25 | 0.27 | 0.27 | 0.29 | 0.31 | 0.31 |
| 366.0 | 0.25 | 0.27 | 0.27 | 0.29 | 0.30 | 0.31 |
| 364.9 | 0.24 | 0.26 | 0.27 | 0.29 | 0.30 | 0.31 |
| 364.0 | 0.24 | 0.26 | 0.26 | 0.29 | 0.30 | 0.31 |
| 362.9 | 0.24 | 0.26 | 0.26 | 0.28 | 0.30 | 0.31 |
| 362.0 | 0.24 | 0.26 | 0.26 | 0.28 | 0.30 | 0.30 |
| 361.1 | 0.24 | 0.26 | 0.26 | 0.28 | 0.30 | 0.31 |
| 360.0 | 0.25 | 0.26 | 0.27 | 0.28 | 0.30 | 0.31 |
| 359.0 | 0.25 | 0.27 | 0.27 | 0.29 | 0.30 | 0.31 |
| 358.0 | 0.25 | 0.27 | 0.27 | 0.29 | 0.30 | 0.31 |
| 357.0 | 0.25 | 0.27 | 0.27 | 0.29 | 0.30 | 0.31 |
| 355.9 | 0.26 | 0.27 | 0.28 | 0.29 | 0.30 | 0.31 |
| 355.0 | 0.27 | 0.28 | 0.28 | 0.30 | 0.31 | 0.31 |
| 354.1 | 0.27 | 0.28 | 0.28 | 0.30 | 0.31 | 0.31 |
| 353.0 | 0.28 | 0.29 | 0.29 | 0.30 | 0.31 | 0.31 |
| 352.0 | 0.28 | 0.29 | 0.29 | 0.30 | 0.31 | 0.31 |
| 351.0 | 0.29 | 0.30 | 0.30 | 0.30 | 0.31 | 0.31 |
| 350.0 | 0.29 | 0.30 | 0.30 | 0.31 | 0.31 | 0.31 |
| 348.9 | 0.30 | 0.30 | 0.30 | 0.31 | 0.31 | 0.31 |
| 348.0 | 0.30 | 0.31 | 0.30 | 0.31 | 0.31 | 0.31 |
| 347.1 | 0.30 | 0.31 | 0.30 | 0.31 | 0.31 | 0.31 |
| 346.0 | 0.30 | 0.31 | 0.30 | 0.31 | 0.31 | 0.31 |
| 345.0 | 0.30 | 0.31 | 0.31 | 0.31 | 0.31 | 0.31 |
| 344.0 | 0.30 | 0.31 | 0.31 | 0.31 | 0.31 | 0.31 |
| 343.0 | 0.30 | 0.31 | 0.30 | 0.31 | 0.31 | 0.31 |
| 341.9 | 0.30 | 0.30 | 0.30 | 0.31 | 0.30 | 0.30 |
| 341.0 | 0.30 | 0.30 | 0.30 | 0.30 | 0.30 | 0.30 |
| 340.1 | 0.30 | 0.30 | 0.30 | 0.30 | 0.30 | 0.30 |
| 339.0 | 0.30 | 0.30 | 0.30 | 0.29 | 0.29 | 0.29 |
| 338.0 | 0.29 | 0.29 | 0.29 | 0.29 | 0.29 | 0.29 |
| 336.9 | 0.29 | 0.29 | 0.29 | 0.29 | 0.29 | 0.28 |
| 336.0 | 0.29 | 0.28 | 0.28 | 0.28 | 0.28 | 0.28 |
| 335.1 | 0.28 | 0.28 | 0.28 | 0.27 | 0.27 | 0.27 |
| 334.0 | 0.27 | 0.27 | 0.27 | 0.27 | 0.27 | 0.27 |
| 333.0 | 0.27 | 0.27 | 0.27 | 0.26 | 0.26 | 0.26 |
| 332.0 | 0.26 | 0.26 | 0.26 | 0.26 | 0.26 | 0.25 |
| 331.0 | 0.25 | 0.26 | 0.25 | 0.25 | 0.25 | 0.25 |
| 329.9 | 0.25 | 0.25 | 0.24 | 0.24 | 0.24 | 0.24 |
| 329.0 | 0.24 | 0.24 | 0.24 | 0.24 | 0.23 | 0.23 |
| 328.1 | 0.23 | 0.23 | 0.23 | 0.23 | 0.23 | 0.23 |
| 327.0 | 0.23 | 0.23 | 0.23 | 0.23 | 0.22 | 0.22 |
| 326.0 | 0.22 | 0.22 | 0.22 | 0.22 | 0.22 | 0.21 |
| 324.9 | 0.21 | 0.21 | 0.21 | 0.21 | 0.21 | 0.21 |
| 324.0 | 0.20 | 0.21 | 0.20 | 0.20 | 0.20 | 0.20 |
| 323.1 | 0.20 | 0.20 | 0.20 | 0.20 | 0.20 | 0.20 |
| 322.0 | 0.19 | 0.20 | 0.19 | 0.19 | 0.19 | 0.19 |
| 321.0 | 0.19 | 0.19 | 0.19 | 0.19 | 0.19 | 0.19 |
| 319.9 | 0.18 | 0.18 | 0.18 | 0.19 | 0.18 | 0.18 |
| 319.0 | 0.18 | 0.18 | 0.18 | 0.18 | 0.18 | 0.18 |
| 318.1 | 0.18 | 0.18 | 0.18 | 0.18 | 0.18 | 0.18 |
| 317.0 | 0.18 | 0.18 | 0.18 | 0.18 | 0.18 | 0.18 |
| 316.0 | 0.18 | 0.18 | 0.18 | 0.18 | 0.18 | 0.18 |
| 314.9 | 0.18 | 0.18 | 0.18 | 0.18 | 0.18 | 0.18 |
| 314.0 | 0.18 | 0.19 | 0.19 | 0.19 | 0.19 | 0.19 |
| 313.1 | 0.19 | 0.19 | 0.19 | 0.19 | 0.19 | 0.19 |
| 312.0 | 0.20 | 0.20 | 0.20 | 0.20 | 0.20 | 0.20 |
| 311.0 | 0.21 | 0.21 | 0.21 | 0.21 | 0.21 | 0.20 |
| 309.9 | 0.22 | 0.22 | 0.22 | 0.22 | 0.22 | 0.22 |

Figure S8, Panel (b) absorbance of 20 µM mb with varying Fe(II) concentrations.

| Fe(II) | absorbance |
| --- | --- |
| 0 | 0.24 |
| 2 | 0.26 |
| 2.5 | 0.26 |
| 5 | 0.28 |
| 7.5 | 0.30 |
| 10 | 0.31 |
| 20 | 0.31 |

Data Table 15. UV-visible absorbance of 26 µM mb in the presence of various Fe(III) concentrations (pH 7.5, 10 mM NaCl) prepared under anoxic conditions. This table shows data from Figure S9.

Figure S9, absorbance of 26 µM mb with varying Fe(III) concentrations.

| wavelength | 0 µM Fe(III) | 5 µM Fe(III) | 10 µM Fe(III) | 20 µM Fe(III) |
| --- | --- | --- | --- | --- |
| 460.1 | 0.01 | 0.01 | 0.01 | 0.02 |
| 459.0 | 0.01 | 0.01 | 0.01 | 0.02 |
| 457.9 | 0.01 | 0.02 | 0.02 | 0.02 |
| 457.0 | 0.01 | 0.02 | 0.02 | 0.02 |
| 455.9 | 0.01 | 0.02 | 0.02 | 0.02 |
| 455.0 | 0.01 | 0.02 | 0.02 | 0.02 |
| 454.0 | 0.01 | 0.02 | 0.02 | 0.02 |
| 453.0 | 0.01 | 0.02 | 0.02 | 0.02 |
| 452.0 | 0.01 | 0.02 | 0.02 | 0.02 |
| 451.0 | 0.02 | 0.02 | 0.02 | 0.03 |
| 450.0 | 0.02 | 0.02 | 0.02 | 0.03 |
| 449.1 | 0.02 | 0.02 | 0.02 | 0.03 |
| 448.0 | 0.02 | 0.02 | 0.02 | 0.03 |
| 447.1 | 0.02 | 0.03 | 0.03 | 0.03 |
| 446.0 | 0.02 | 0.03 | 0.03 | 0.03 |
| 445.1 | 0.02 | 0.03 | 0.03 | 0.03 |
| 444.0 | 0.03 | 0.03 | 0.03 | 0.04 |
| 442.9 | 0.03 | 0.03 | 0.03 | 0.04 |
| 442.0 | 0.03 | 0.04 | 0.04 | 0.04 |
| 440.9 | 0.03 | 0.04 | 0.04 | 0.04 |
| 440.0 | 0.04 | 0.04 | 0.04 | 0.05 |
| 438.9 | 0.04 | 0.05 | 0.05 | 0.05 |
| 438.0 | 0.04 | 0.05 | 0.05 | 0.05 |
| 436.9 | 0.05 | 0.05 | 0.06 | 0.06 |
| 436.0 | 0.05 | 0.06 | 0.06 | 0.06 |
| 435.0 | 0.06 | 0.07 | 0.07 | 0.07 |
| 434.0 | 0.07 | 0.07 | 0.07 | 0.08 |
| 433.0 | 0.07 | 0.08 | 0.08 | 0.08 |
| 432.0 | 0.08 | 0.08 | 0.08 | 0.09 |
| 431.0 | 0.09 | 0.09 | 0.09 | 0.09 |
| 430.0 | 0.09 | 0.10 | 0.10 | 0.10 |
| 429.0 | 0.11 | 0.11 | 0.11 | 0.11 |
| 428.0 | 0.11 | 0.12 | 0.12 | 0.12 |
| 427.0 | 0.12 | 0.13 | 0.13 | 0.13 |
| 426.0 | 0.13 | 0.14 | 0.14 | 0.14 |
| 425.0 | 0.14 | 0.15 | 0.15 | 0.15 |
| 424.0 | 0.16 | 0.16 | 0.16 | 0.16 |
| 423.0 | 0.17 | 0.18 | 0.18 | 0.18 |
| 422.0 | 0.18 | 0.19 | 0.19 | 0.19 |
| 421.0 | 0.19 | 0.20 | 0.20 | 0.20 |
| 420.0 | 0.21 | 0.21 | 0.21 | 0.21 |
| 419.0 | 0.22 | 0.23 | 0.23 | 0.23 |
| 418.0 | 0.24 | 0.24 | 0.25 | 0.24 |
| 417.0 | 0.25 | 0.26 | 0.26 | 0.25 |
| 416.0 | 0.26 | 0.27 | 0.27 | 0.27 |
| 415.0 | 0.28 | 0.29 | 0.29 | 0.28 |
| 414.0 | 0.30 | 0.30 | 0.30 | 0.30 |
| 413.0 | 0.31 | 0.32 | 0.32 | 0.31 |
| 412.0 | 0.33 | 0.33 | 0.33 | 0.33 |
| 411.0 | 0.34 | 0.34 | 0.35 | 0.34 |
| 410.0 | 0.35 | 0.36 | 0.36 | 0.35 |
| 408.9 | 0.37 | 0.37 | 0.37 | 0.37 |
| 408.0 | 0.38 | 0.39 | 0.39 | 0.38 |
| 406.9 | 0.39 | 0.40 | 0.40 | 0.39 |
| 406.0 | 0.40 | 0.41 | 0.41 | 0.40 |
| 404.9 | 0.41 | 0.42 | 0.42 | 0.41 |
| 404.0 | 0.42 | 0.43 | 0.43 | 0.42 |
| 402.9 | 0.43 | 0.44 | 0.44 | 0.43 |
| 402.0 | 0.44 | 0.44 | 0.45 | 0.43 |
| 400.9 | 0.44 | 0.45 | 0.45 | 0.44 |
| 400.0 | 0.45 | 0.46 | 0.46 | 0.44 |
| 399.1 | 0.45 | 0.46 | 0.46 | 0.45 |
| 398.0 | 0.46 | 0.46 | 0.47 | 0.45 |
| 397.1 | 0.46 | 0.47 | 0.47 | 0.46 |
| 396.0 | 0.46 | 0.47 | 0.47 | 0.46 |
| 395.1 | 0.46 | 0.47 | 0.47 | 0.46 |
| 394.0 | 0.46 | 0.47 | 0.47 | 0.46 |
| 393.0 | 0.46 | 0.47 | 0.47 | 0.46 |
| 392.0 | 0.46 | 0.47 | 0.47 | 0.46 |
| 391.0 | 0.45 | 0.46 | 0.47 | 0.45 |
| 390.0 | 0.45 | 0.46 | 0.46 | 0.45 |
| 389.0 | 0.45 | 0.46 | 0.46 | 0.45 |
| 387.9 | 0.44 | 0.45 | 0.45 | 0.44 |
| 387.0 | 0.44 | 0.44 | 0.45 | 0.44 |
| 385.9 | 0.43 | 0.44 | 0.44 | 0.43 |
| 385.0 | 0.42 | 0.43 | 0.44 | 0.43 |
| 384.1 | 0.42 | 0.43 | 0.43 | 0.42 |
| 383.0 | 0.41 | 0.42 | 0.42 | 0.41 |
| 382.1 | 0.40 | 0.41 | 0.41 | 0.40 |
| 381.0 | 0.39 | 0.40 | 0.40 | 0.40 |
| 380.0 | 0.38 | 0.39 | 0.40 | 0.39 |
| 379.0 | 0.38 | 0.39 | 0.39 | 0.39 |
| 378.0 | 0.37 | 0.38 | 0.38 | 0.38 |
| 376.9 | 0.36 | 0.37 | 0.37 | 0.37 |
| 376.0 | 0.35 | 0.36 | 0.36 | 0.36 |
| 374.9 | 0.34 | 0.35 | 0.35 | 0.35 |
| 374.0 | 0.34 | 0.34 | 0.35 | 0.35 |
| 373.1 | 0.33 | 0.34 | 0.34 | 0.34 |
| 372.0 | 0.32 | 0.33 | 0.33 | 0.33 |
| 371.1 | 0.32 | 0.33 | 0.33 | 0.33 |
| 370.0 | 0.31 | 0.32 | 0.32 | 0.32 |
| 369.0 | 0.31 | 0.31 | 0.32 | 0.32 |
| 368.0 | 0.30 | 0.31 | 0.31 | 0.32 |
| 367.0 | 0.29 | 0.30 | 0.31 | 0.31 |
| 365.9 | 0.29 | 0.30 | 0.31 | 0.30 |
| 365.0 | 0.29 | 0.30 | 0.31 | 0.31 |
| 364.1 | 0.29 | 0.30 | 0.30 | 0.30 |
| 363.0 | 0.29 | 0.30 | 0.30 | 0.31 |
| 362.1 | 0.29 | 0.30 | 0.30 | 0.31 |
| 361.0 | 0.29 | 0.30 | 0.31 | 0.31 |
| 360.0 | 0.30 | 0.31 | 0.31 | 0.32 |
| 358.9 | 0.31 | 0.31 | 0.32 | 0.32 |
| 358.0 | 0.31 | 0.32 | 0.33 | 0.33 |
| 356.9 | 0.32 | 0.33 | 0.34 | 0.34 |
| 356.0 | 0.33 | 0.34 | 0.34 | 0.35 |
| 355.1 | 0.34 | 0.34 | 0.35 | 0.35 |
| 354.0 | 0.35 | 0.35 | 0.36 | 0.37 |
| 353.0 | 0.36 | 0.36 | 0.37 | 0.37 |
| 352.0 | 0.37 | 0.38 | 0.38 | 0.38 |
| 351.0 | 0.38 | 0.38 | 0.39 | 0.39 |
| 349.9 | 0.38 | 0.39 | 0.40 | 0.40 |
| 349.0 | 0.39 | 0.40 | 0.41 | 0.40 |
| 348.1 | 0.40 | 0.40 | 0.41 | 0.41 |
| 347.0 | 0.40 | 0.41 | 0.42 | 0.42 |
| 346.0 | 0.40 | 0.41 | 0.42 | 0.42 |
| 344.9 | 0.41 | 0.41 | 0.42 | 0.43 |
| 344.0 | 0.41 | 0.42 | 0.43 | 0.43 |
| 342.9 | 0.41 | 0.42 | 0.43 | 0.43 |
| 342.0 | 0.41 | 0.42 | 0.43 | 0.43 |
| 341.1 | 0.41 | 0.42 | 0.43 | 0.43 |
| 340.0 | 0.40 | 0.42 | 0.42 | 0.42 |
| 339.0 | 0.40 | 0.42 | 0.42 | 0.42 |
| 337.9 | 0.40 | 0.41 | 0.42 | 0.42 |
| 337.0 | 0.40 | 0.41 | 0.42 | 0.42 |
| 336.1 | 0.39 | 0.40 | 0.41 | 0.41 |
| 335.0 | 0.38 | 0.39 | 0.40 | 0.41 |
| 334.0 | 0.38 | 0.39 | 0.40 | 0.40 |
| 332.9 | 0.37 | 0.38 | 0.39 | 0.39 |
| 332.0 | 0.36 | 0.37 | 0.38 | 0.39 |
| 330.9 | 0.35 | 0.36 | 0.37 | 0.38 |
| 330.0 | 0.34 | 0.35 | 0.36 | 0.37 |
| 329.1 | 0.34 | 0.35 | 0.35 | 0.36 |
| 328.0 | 0.33 | 0.34 | 0.35 | 0.35 |
| 327.0 | 0.32 | 0.33 | 0.34 | 0.35 |
| 325.9 | 0.31 | 0.32 | 0.33 | 0.34 |
| 325.0 | 0.30 | 0.31 | 0.32 | 0.32 |
| 324.1 | 0.29 | 0.30 | 0.31 | 0.32 |
| 323.0 | 0.28 | 0.29 | 0.30 | 0.31 |
| 322.0 | 0.27 | 0.29 | 0.30 | 0.31 |
| 320.9 | 0.27 | 0.28 | 0.29 | 0.30 |
| 320.0 | 0.26 | 0.28 | 0.29 | 0.30 |
| 319.1 | 0.26 | 0.27 | 0.28 | 0.30 |
| 318.0 | 0.26 | 0.27 | 0.28 | 0.29 |
| 317.0 | 0.26 | 0.27 | 0.28 | 0.30 |
| 315.9 | 0.26 | 0.27 | 0.28 | 0.30 |
| 315.0 | 0.27 | 0.28 | 0.29 | 0.30 |
| 314.1 | 0.27 | 0.28 | 0.29 | 0.31 |
| 313.0 | 0.28 | 0.29 | 0.31 | 0.32 |
| 312.0 | 0.30 | 0.31 | 0.32 | 0.33 |
| 310.9 | 0.31 | 0.32 | 0.33 | 0.35 |
| 310.0 | 0.33 | 0.34 | 0.35 | 0.37 |
